# Induction of cell cycle arrest and inflammatory genes by combined treatment with epigenetic, differentiating, and chemotherapeutic agents in triple-negative breast cancer

**DOI:** 10.1186/s13058-018-1068-x

**Published:** 2018-11-28

**Authors:** Vanessa F. Merino, Soonweng Cho, Nguyen Nguyen, Helen Sadik, Athira Narayan, Conover Talbot, Leslie Cope, Xian C. Zhou, Zhe Zhang, Balázs Győrffy, Saraswati Sukumar

**Affiliations:** 1 Department of Radiology, Johns Hopkins University School of Medicine, Baltimore, MD USA; 2 Department of Neuroscience, Johns Hopkins University School of Medicine, Baltimore, MD USA; 3 Department of Oncology, Johns Hopkins University School of Medicine, Baltimore, MD USA; 4 MTA TTK Lendület Cancer Biomarker Research Group, Institute of Enzymology, Budapest, Hungary; 5 2nd Department of Pediatrics, Semmelweis University, Budapest, Hungary

**Keywords:** Breast cancer, Entinostat, Epigenetic, Immunotherapy

## Abstract

**Background:**

A combination of entinostat, all-trans retinoic acid, and doxorubicin (EAD) induces cell death and differentiation and causes significant regression of xenografts of triple-negative breast cancer (TNBC).

**Methods:**

We investigated the mechanisms underlying the antitumor effects of each component of the EAD combination therapy by high-throughput gene expression profiling of drug-treated cells.

**Results:**

Microarray analysis showed that entinostat and doxorubicin (ED) altered expression of genes related to growth arrest, inflammation, and differentiation. ED downregulated MYC, E2F, and G2M cell cycle genes. Accordingly, entinostat sensitized the cells to doxorubicin-induced growth arrest at G2. ED induced interferon genes, which correlated with breast tumors containing a higher proportion of tumor-infiltrating lymphocytes. ED also increased the expression of immune checkpoint agonists and cancer testis antigens. Analysis of TNBC xenografts showed that EAD enhanced the inflammation score in nude mice. Among the genes differentially regulated between the EAD and ED groups, an all-trans retinoic acid (ATRA)-regulated gene, DHRS3, was induced in EAD-treated xenografts. DHRS3 was expressed at lower levels in human TNBC metastases compared to normal breast or primary tumors. High expression of ED-induced growth arrest and inflammatory genes was associated with better prognosis in TNBC patients.

**Conclusions:**

Entinostat potentiated doxorubicin-mediated cell death and the combination induced inflammatory signatures. The ED-induced immunomodulation may improve immunotherapy. Addition of ATRA to ED may potentiate inflammation and contribute to TNBC regression.

**Electronic supplementary material:**

The online version of this article (10.1186/s13058-018-1068-x) contains supplementary material, which is available to authorized users.

## Background

All-trans retinoic acid (ATRA) induces breast cancer differentiation [1, 2], cell death [3], and inflammation [4]. However, its limited treatment success in solid tumors [5] may be attributed to the frequent epigenetic silencing of the retinoic acid receptor beta (*RAR-β*) [6, 7]. We and others have shown that *RAR-β* is underexpressed and epigenetically silenced in breast cancer, and that histone deacetylase inhibitors (HDACi) cause reexpression of *RAR-β* and sensitize the cells to treatment [8, 9].

HDACi are currently employed in the clinic for the treatment of a wide variety of solid and hematological malignancies [10], including breast cancer [11]. The proposed anticancer activities of HDACi include: the induction of apoptosis and cell cycle arrest [12, 13], the inhibition of angiogenesis [14, 15], and the stimulation of cancer cell differentiation [16]. Importantly, HDACi have been shown to enhance the immunogenicity of cancer cells [17].

Although HDACi showed limited effect as single agents to treat breast cancer, their use in combination with other anticancer agents is currently being evaluated [11]. Studies in advanced solid tumors in which HDACi were combined either with doxorubicin [18] or retinoic acid [19] suggested enhanced antitumor activity. Previously, we showed that a combination of entinostat, ATRA, and low-dose doxorubicin (EAD) effectively induced cell death and differentiation and decreased tumor size in xenografts of TNBC cell lines [20]. In this study, we provide insights into additional mechanisms underlying the combined drug effect on the decrease of tumor volume. Entinostat and doxorubicin combination (ED) induced cell cycle growth arrest, interferon response, and other inflammatory signatures. Addition of ATRA to the entinostat and doxorubicin combination (EAD) most effectively induced inflammatory features in nude mice.

## Methods

### Patient samples, cell lines, constructs, and reagents

RNA and cDNA were generated from normal and breast cancer tissue, as described previously [20]. MDA-MB-231 and HCC1937 cells were obtained from the American Type Culture Collection and SUM-149 and SUM-159 cells were obtained from Dr. S. Ethier (Wayne State University, Detroit, MI, USA). ATRA and doxorubicin were purchased from Sigma Chemicals. Entinostat was provided by Syndax Pharmaceuticals, LLC.

### Transcriptome array and bioinformatics analysis

MDA-MB-231 cells were treated for 48 h with entinostat (2.5 μM), ATRA (1 μM), and doxorubicin (0.2 μM) singly or in combination. RNA was extracted using RNeasy Mini Kit (Qiagen) and Illumina HumanHT12v4 gene expression array was performed by the Microarray Core at Johns Hopkins. Illumina microarray data were preprocessed by background subtraction followed by quantile normalization using GenomeStudio. After preprocessing using GenomeStudio, data were imported and analyzed using R (using base and Bioconductor packages) and Ingenuity® Pathway Analysis (IPA). Detailed methods are provided in Additional file 1.

### Fluorescence-activated cell quantification

For cell cycle determinations, cells were permeabilized with cold 70% ethanol and stained with propidium iodide (Sigma). Samples were run on the BD FACSCalibur system (Becton Dickinson).

### Xenograft assay

Xenografts of MDA-MB-231 cells were established in six to eight athymic nude mice by injecting 2 × 10^6^ tumor cells per flank, two flanks per mouse. The mice were treated for 4 weeks, receiving entinostat (2.5 mg/kg) for 5 days/weeks oral, ATRA (5 mg/kg) 5 days/weeks i.p., and doxorubicin (2 mg/kg) once a week i.v.

### Correlation of gene expression and patient outcome

Kaplan–Meier analysis was performed by utilizing an extended version of the previously established breast cancer transcriptomic database [21]. The Kaplan–Meier survival plot, the hazard ratio with 95% confidence intervals, and the logrank *p* value were calculated and plotted in R using the package “survival” [22].

### Statistical analysis

The cell line results were expressed as mean ± standard errors of mean (SEM). Two-tailed Student’s *t* tests (95% confidence interval) were performed on pairwise combinations of data to determine statistical significance defined as *p* < 0.05, *p* < 0.01, and *p* < 0.001. Results of qRT-PCR using tumor xenograft cDNAs were analyzed using the median and two-tailed Mann–Whitney test. Statistical analyses were performed using GraphPad Prism version 5.0 (GraphPad Software, Inc.).

## Results

### Entinostat and doxorubicin combination induces gene reprogramming

Previously, we showed that a combination of entinostat, ATRA, and doxorubicin (EAD) resulted in increased cell death and differentiation, and as a consequence, a decrease of tumor volume [20]. To explore additional mechanisms of tumor regression caused by EAD, and to identify drug targets other than RAR-β, we performed high-throughput gene expression profiling analysis of MDA-MB-231 cells treated with entinostat, ATRA, and doxorubicin as single, double, and triple combinations.

**Fig. 1:**
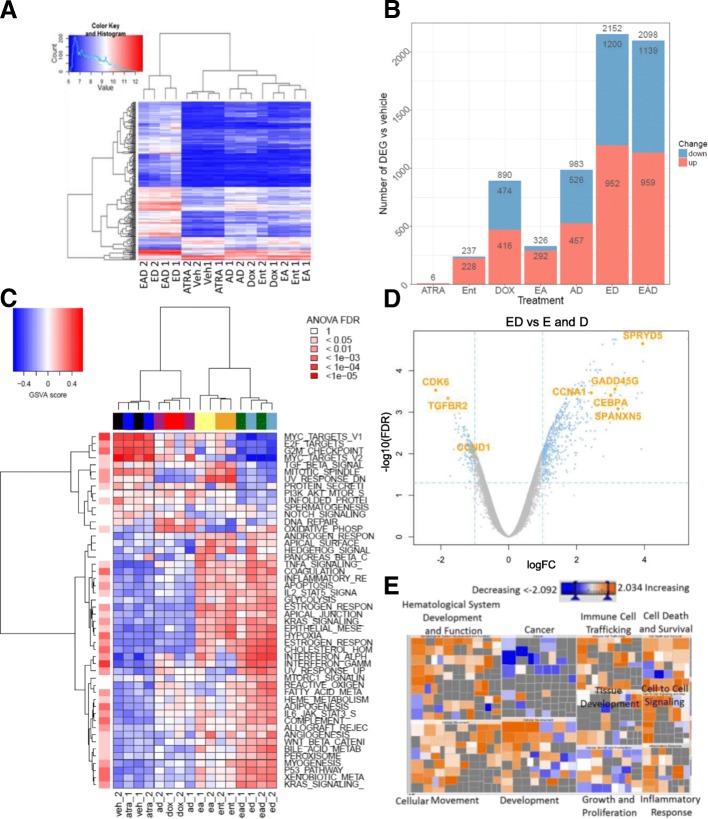
ED regulates inflammatory, arrest, and differentiation genes. **a** Heatmap depicting unsupervised hierarchical clustering of top 10% differentially expressed genes in MDA-MB-231 cells following different treatments (2.5 μM entinostat, 1 μM ATRA, 0.2 μM doxorubicin). Color scale indicates log2 expression values. **b** Total number of genes regulated in MDA-MB-231 cells by indicated treatments. **c** Gene set variation analysis (GSVA) scores of gene set analysis (GSEA) hallmark gene sets for samples in the study. Heatmap colors from blue to red represent low to high enrichment; red color bar (right) shows false discovery rate (FDR)-adjusted *p* value from one-way analysis of variance (ANOVA) test comparing GSVA enrichment scores across treatment groups. **d** Volcano plot (log2 fold-change (FC) vs – log10 FDR) of genes upregulated and downregulated in MDA-MB-231 cells following treatment with ED in comparison to cells treated singly with E or D. Orange dots represent genes with at least 2-fold increase or decrease in gene expression and FDR < 0.05. **e** Heatmap of disease and biological functions analysis using Ingenuity® Pathway Analysis software on ED genes. Size and color of the boxes represent – log *p* value and *z* score, respectively. E entinostat (Ent), A all-trans retinoic acid (ATRA), D doxorubicin (Dox), Veh vehicle

An unsupervised cluster analysis using the top 10% most differentially expressed genes across all eight different treatment conditions revealed clustering of vehicle with ATRA and ED with EAD treatment groups (Fig. 1a). Principal component analysis (PCA) revealed similar grouping of samples on the first component and a minor batch effect on the second component (Additional file 2: Figure S1A) which was corrected in further analysis.

Differential expression analysis identified gene expression changes (≥ 2-fold change and false discovery rate ≤ 0.05) across all seven different combinations compared to vehicle treatment (Fig. 1b). EAD and ED combination treatments elicited the greatest number of differentially expressed genes, at 2098 and 2152 respectively. Consistent with the behavior of HDAC inhibitors, most of the genes altered by entinostat treatment alone are upregulated (228/237, or 96.2%).

### Gene set analysis reveals independent and synergistic biological roles for each treatment modality

A comprehensive analysis of the pathways regulated by the treatment groups in comparison to vehicle was performed using gene set variant analysis (GSVA) [23] on Hallmark gene sets defined by the Molecular Signatures Database (MSigDB). ANOVA with multiple test correction was performed for each gene set across all treatment groups to identify differentially regulated gene sets (Fig. 1c).

ATRA treatment alone had a gene set profile similar to vehicle while doxorubicin downregulated genes related to mitotic spindle and UV response. As described previously for other HDACi [24], entinostat increased expression of genes associated with angiogenesis, as well as genes related to TNF-α, inflammatory response, apoptosis, early estrogen receptor (ER) response, and others (Fig. 1c). Doxorubicin and entinostat single treatments downregulated MYC and E2F targets and progression through the G2M checkpoint, in comparison to vehicle (Fig. 1c). However, the ED/EAD combination most effectively decreased expression of these genes related to proliferation and cell-cycle progression. ED/EAD also downregulated genes related to TGF-β, protein secretion, and unfolded protein response. Interestingly, ED/EAD combinations most effectively upregulated IFN-α, IFN-γ, complement, TP53, and UV-response genes (Fig. 1c). Although some of these latter pathways were also upregulated to a lesser extent by doxorubicin or entinostat, IFN-α and IFN-γ showed the strongest synergistic enrichment following treatment with ED (Fig. 1c).

### Combined entinostat and doxorubicin regulates growth arrest, inflammation, and differentiation pathways

Due to the large gene expression effect of ED treatment, we focused our analysis on identifying differences between ED versus single treatments with entinostat or doxorubicin. In this analysis, 868 genes were differentially expressed (Additional file 2: Figure S1B, Additional file 3: Table S1, and Additional file 4: Table S2), including upregulation of cell cycle, interferon, and CTA genes and downregulation of cell cycle genes (Fig. 1d), which suggests a synergistic effect of ED combination treatment on inducing cell death and inflammation (Fig. 1c).

To determine the activation of specific biological functions potentially related to a decrease of tumor volume due to EAD treatment, we performed pathway enrichment analysis using Ingenuity® Pathway Analysis (IPA) on the 868 synergistically differentially expressed genes following ED treatment (Fig. 1e). Cancer-related pathways showed negative scores, while inflammatory response (Additional file 1: Figure S1C), cellular movement (Additional file 2: Figure S1D), and immune cell trafficking pathways had positive scores (Fig. 1e and Additional file 5: Table S3). Other pathways related to cell death and survival, growth and proliferation, and development were also identified, with mixed enrichment scores (Fig. 1e and Additional file 5: Table S3).

Consistent with IPA analysis, gene set enrichment analysis (GSEA) identified increased inflammation-related (interferon alpha and gamma), cell death (upregulation of TP53, apoptosis), and growth arrest and proliferation (decreased G2M progression, E2F and myc targets) gene sets in the ED gene signature (Additional file 6: Table S4).

### ED and EAD therapies induce cell growth arrest

**Fig. 2:**
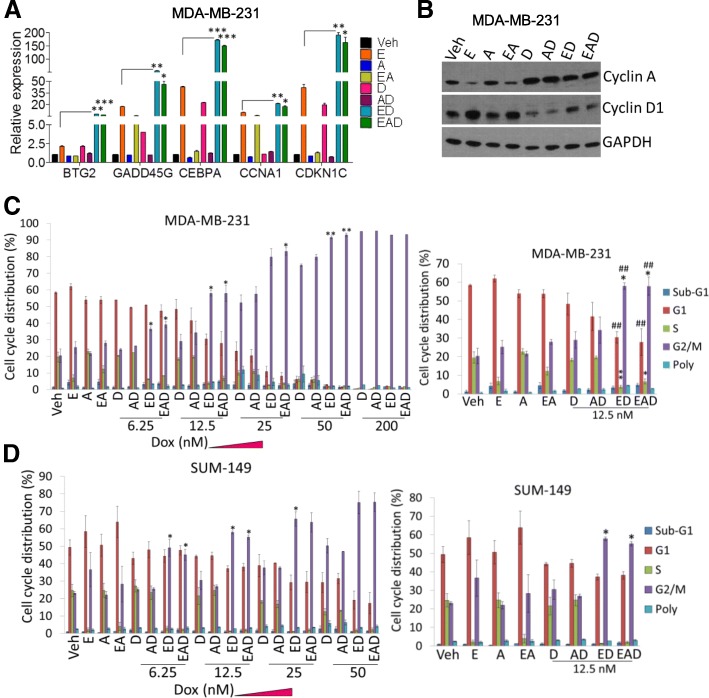
ED and EAD induce growth arrest. **a** qRT-PCR of few genes, related to cell growth arrest and death (identified by array analysis), in MDA-MB-231 cells treated with entinostat (2.5 μM), ATRA (1 μM), and doxorubicin (0.2 μM) singly, and combinations, for 48 h. **b** Western blot analysis of cyclin A and cyclin D1 on MDA-MB-231 cells, treated as described in text. Flow cytometry determination of percentage of cell cycle distribution of (**c**) MDA-MB-231 and (**d**) SUM-149 cells treated with different groups containing doxorubicin 6.25–200 nM (doxorubicin 12.5 nM highlighted on right), for 48 h. *RPL39* and GAPDH used as controls for qRT-PCR and western blot, respectively. Student’s *t* test performed, mean (± SEM) of triplicate results shown. *Compared to entinostat in qRT-PCR and doxorubicinin flow cytometry; #compared to entinostat in flow cytometry: *p < 0.05, **/##*p* < 0.01, ***p < 0.001. E entinostat, A all-trans retinoic acid, D doxorubicin (Dox), Veh vehicle, GAPDH glyceraldehyde 3-phosphate dehydrogenase

Among the genes related to cell growth arrest and death identified by array analysis, we validated the following ED upregulated genes by qRT-PCR: *BTG2*, *GADD45G*, *CEBPA*, *CCNA1*, and *CDKN1C*. Their mRNA expression levels following ED treatment were 2–4.5-fold higher than entinostat-treated MDA-MB-231 cells (Fig. 2a). In contrast to the decrease of *cyclin D1 *by entinostat, observed mainly in ER-positive cell lines [25], treatment of TNBC cells with entinostat induced *cyclin D1* and its target, *CDK6*, mRNA in comparison to vehicle-treated cells (Additional file 7: Figure S2A). However, ED and EAD treatment significantly decreased *cyclin D1* and *CDK6* mRNA, in comparison to entinostat (Additional file 7: Figure S2A). Similarly, cyclin-D1 protein levels were higher in entinostat-treated cells, and significantly decreased by doxorubicin, present in ED and EAD (Fig. 2b and Additional file 7: Figure S2B, left). On the other hand, the level of the tumor suppressor cyclin-A (CCNA1) protein was decreased by entinostat treatment, and restored by doxorubicin in ED and EAD (Fig. 2b and Additional file 7: Figure S2B, right panel).

Previously, we reported that ED therapy induces cell death by an increase in apoptosis in cell culture and in tumor xenografts [20]. EAD most effectively induced apoptosis and necrosis and cell death [20]. Since we observed an increase in cell growth arrest genes by ED and EAD treatment (Fig. 2a, b), we further analyzed whether cell growth arrest is involved in the ED and EAD-induced cell death. As expected, high doses of doxorubicin (200 nM) induced arrest at the G2 phase of the cell cycle (Fig. 2c). Interestingly, ED increased arrest at the G2 phase in comparison to doxorubicin alone (6.25–50 nM) in MDA-MB-231 cells (Fig. 2c), SUM-149 cells (Fig. 2d), and HCC1937 cells (Additional file 8: Table S5), suggesting that entinostat increases doxorubicin sensitivity and growth arrest. Entinostat also significantly decreased the S phase of the cell cycle in the three cell lines (Fig. 2c, d and Additional file 8: Table S5), consistent with its effect on decreasing cell proliferation [20].

### ED and EAD induce interferon genes associated with higher immune cell infiltration

**Fig. 3:**
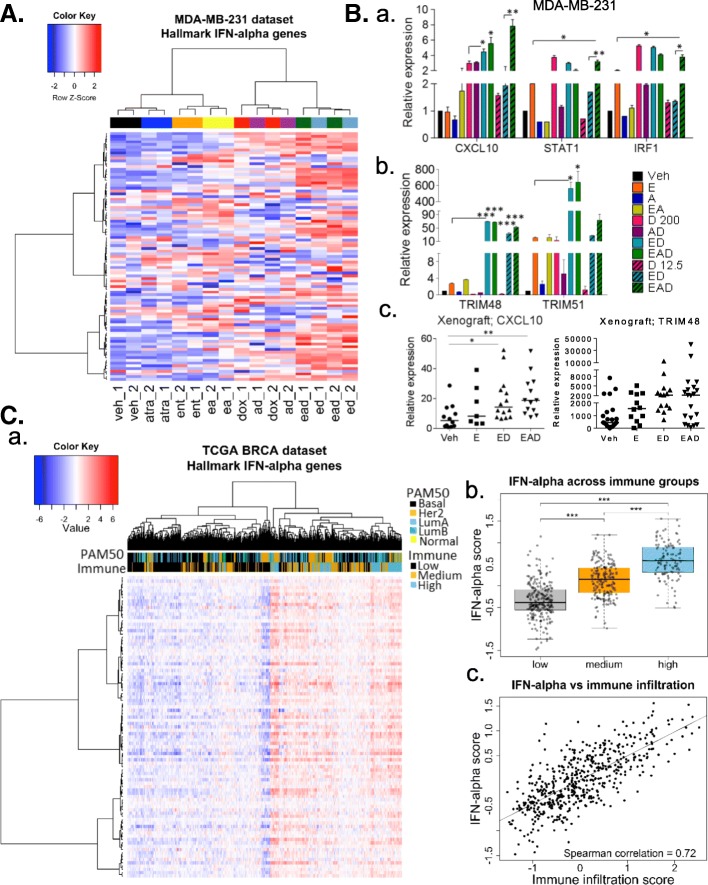
ED and EAD induce expression of interferon-alpha genes. (**A**) Hierarchical supervised clustering of expression of *interferon (IFN)-α* genes against signatures of MDA-MB-231 cells following treatments. (**B**) qRT-PCR of (a) type 1 *IFN *genes (*CXCL10, STAT1 and IRF1*) and (b) interferon-responsive genes (*TRIM48* and *TRIM51*) in MDA-MB-231 cells and (c) *CXCL10* and *TRIM48* in xenografts of treated mice. *t* test used to compare mean level of expression (± SEM) in qRT-PCR after *RPL39* normalization. *Compared to entinostat or doxorubicin: *p < 0.05, **p < 0.01, ***p < 0.001. Mann–Whitney test performed, median of *CXCL10* and *TRIM48* expression in xenografts shown. (**C**) (a) Hierarchical supervised clustering of expression of IFN-α genes against The Cancer Genome Atlas (TCGA) RNA-seq breast cancer patient dataset (bars above identify different tumor subtypes (PAM50) and inflammatory cell content (immune, low–high)); (b) one-way ANOVA showed significant difference across one or more groups (#1 low, #2 medium, #3 high immune cells) and post-hoc pairwise Student *t* test with Bonferroni correction revealed statistically significant differences across all groups (*p* < 0.05); (c) IFN-α score correlation with immune infiltration. E entinostat (Ent), A all-trans retinoic acid (ATRA), D doxorubicin (Dox), Veh vehicle, Her2 human epidermal growth factor receptor 2, Lum luminal

Since we identified regulation of interferon (IFN) response genes as one of the most significant pathways associated with ED treatment (Fig. 1c and Additional file 6: Table S4), we analyzed the expression of type-1 IFN genes in the array, and validated expression by qRT-PCR. Hierarchical clustering analysis of these gene sets revealed upregulation of IFN-α (Fig. 3A and Additional file 9: Table S6) and IFN-γ (Additional file 10: Figure S3A and Additional file 11: Table S7) genes by ED and EAD in comparison to doxorubicin and the other groups in MDA-MB-231 cells. Within the IFN genes, *GBP1*, *CXCL10*, *IRF1*, and *STAT1* (Fig. 3A) were described previously to be part of a tumor relapse-free signature in breast cancer patients [26]. By qRT-PCR, we showed that *CXCL10*, *IRF1*, and *STAT1* were induced in MDA-MB-231 by EAD (at low doses of doxorubicin), in comparison to ED and single treatments (Fig. 3Ba). Interferon-responsive genes, the tripartite motif (TRIM) proteins *TRIM48* and *TRIM51* (Fig. 3Bb), and interferon gamma (*IFNG*) (Additional file 10: Figure S3Ba) were induced at higher levels in MDA-MB-231 cells by ED and EAD treatments in comparison to single treatments. We observed a significant increase of *CXCL10* and a trend to increase *TRIM48* expression in mice xenografts of MDA-MB-231 cells treated with ED and EAD (Fig. 3Bc). The induction of proinflammatory genes such as *CXCL10* and *TRIM48* by epigenetic therapy was also observed in another TNBC cell line, SUM-159 (Additional file 10: Figure S3Bb).

Lastly, we sought to identify the relationship of immune infiltration in breast cancer with respect to the expression of IFN-α (Fig. 3A) and IFN-γ (Additional file 10: Figure S3A) genes induced by ED. Immune infiltration groups were identified in The Cancer Genome Atlas (TCGA) breast cancer dataset using a core set of immune genes [27] into clusters of low, medium, and high infiltration markers (Additional file 10: Figure S3C). Hierarchical clustering was performed in the same TCGA samples using these ED-induced IFN genes. There was a significant enrichment of samples with high immune infiltration in a cluster with high expression of ED-induced IFN genes, with stepwise decrease of immune infiltration groups with lower ED-induced IFN gene expression (Fig. 3Ca and Additional file 10: Figure S3D, Additional file 9: Table S6, and Additional file 11: Table S7). There is a strong correlation between ED-induced IFN-α (Fig. 3Cb, Cc) and IFN-γ (Additional file 10: Figure S3E, F) gene scores and immune infiltration scores (Spearman correlation = 0.72 and 0.85, respectively). A 10,000-fold gene-wise permutation analysis to identify significant association between immune infiltration genes and IFN genes revealed statistically significant association (*p* < 0.001) (Fig. 3Cb, Cc and Additional file 10: Figure S3E, F). These analyses suggest that the increase of IFN genes by ED and EAD treatment may increase the infiltration of immune cells into the breast tumor site.

### ED induces proinflammatory genes

**Fig. 4:**
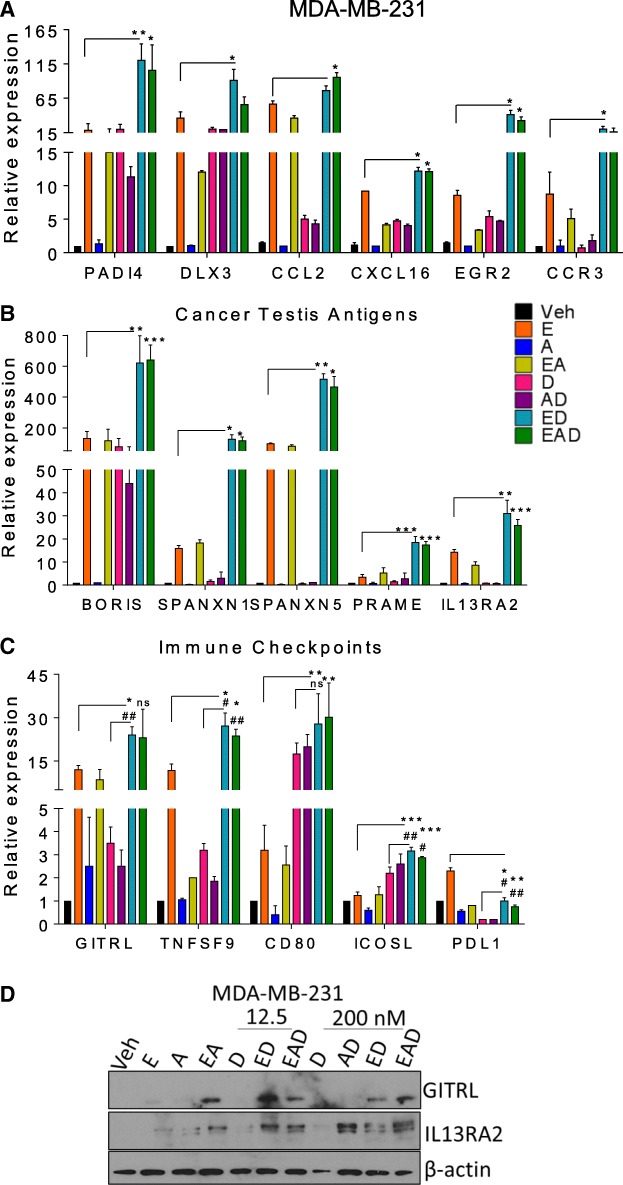
ED and EAD induce expression of proinflammatory genes. qRT-PCR of (**a**) genes associated with immune cell activation and recruitment, (**b**) cancer testis antigens (CTA), and (**c**) immune checkpoints. *t* test used to compare mean level of expression (± SEM) in qRT-PCR after *RPL39* normalization. *Compared to entinostat; #compared to doxorubicin: */#*p* < 0.05, **/##*p* < 0.01, ***p < 0.001. **d** Western blot analysis of GITRL and IL13RA2 in MDA-MB-231 cells treated as described in text. Loading control: β-actin. E entinostat, A all-trans retinoic acid, D doxorubicin, Veh vehicle, ns not significant

In addition, we identified by array analysis that ED regulated different inflammatory genes, including the *CCL2*, *CXCL16* (Fig. 4a), and *TNFSF9* (Fig. 4c) cytokines, the *EGR2* and *DLX3* transcription factors (Fig. 4a), the *PADI4* enzyme (Fig. 4a), the *CCR3* receptor (Fig 4a), and the *SPANXN1* and *SPANXN5 *cancer testis antigens (CTAs) (Fig. 4b), which are related to immune cells recruitment and activation (Additional file 2: Figure S1C, D). qRT-PCR validation showed that entinostat induced the expression of these inflammatory genes in MDA-MB-231 cells and this effect is further potentiated by ED (Fig. 4). Since we observed the ED induction of the CTAs *SPANXN5* in MDA-MB-231 cells (Fig. 4b) and *SPANXN1* in both MDA-MB-231 (Fig. 4b) and SUM-159 cells (Additional file 12: Figure S4A), and the immune checkpoint agonist *TNFSF9/CD137L* in MDA-MB-231 cells (Fig. 4c) in the array and qRT-PCR, we studied the expression of other members of these gene families across our treatment groups.

The CTAs *CTCFL*/*BORIS*, *IL13RA2*, and *PRAME* are significantly induced by entinostat and further regulated by the combination of entinostat and doxorubicin (ED) (Fig. 4b). ED also induced the expression of different checkpoint agonists, including the ligands *ICOSL *and *GITRL* (Fig. 4c) in MDA-MB-231 cells. The immune checkpoint *CD80* is also induced by entinostat, and further regulated by doxorubicin. The ligand *PDL1* is induced by entinostat and significantly decreased by doxorubicin (Fig. 4c). The checkpoint agonist GITRL and the cancer testis antigen IL13RA2 proteins were also induced in MDA-MB-231 cells by ED and EAD treatment (Fig. 4d and Additional file 12: Figure S4B). These patterns suggest activation of the immune system by ED as possible contributors to tumor cell death in an in-vivo system.

### EAD combination induces inflammatory features in xenografts in nude mice

**Fig. 5:**
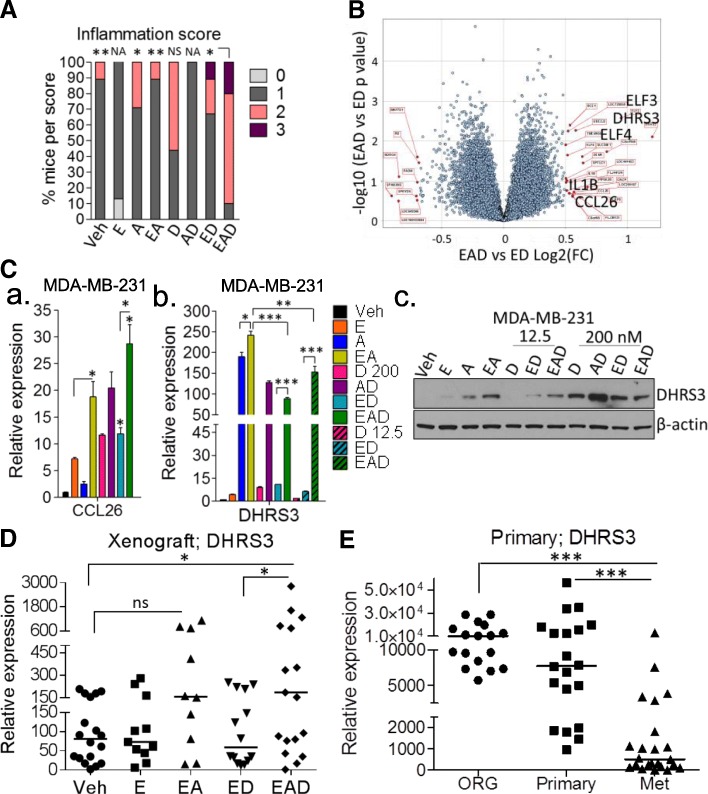
EAD induces inflammatory features and DHRS3 expression. (**A**) Quantification of inflammation from tumor xenografts (*n* = 7–10/group) of treated mice (score 0–3) by pathologist blinded to nature of treatment. (**B**) Volcano plot (log2 fold-change (FC) vs – log10 *p* value) of genes upregulated and downregulated in MDA-MB-231 cells following EAD in comparison to ED treatments. (**C**) qRT-PCR detection of (a) *CCL26* and (b) *DHRS3* in MDA-MB-231 cells treated with entinostat (2.5 μM), ATRA (1 μM), and doxorubicin (200 and 12.5 nM) singly, and combinations, for 48 h; (c) western blot analysis of DHRS3 in MDA-MB-231 cells treated as described in text. Loading control: β-actin. qRT-PCR detection of *DHRS3* mRNA levels in (**D**) tumor xenografts treated as indicated and (**E**) normal breast organoids (ORG), primary, and metastatic samples from TNBC patients. Mann–Whitney test performed, median of *DHRS3* expression in xenograft and primary samples shown. Student’s *t* test performed, mean (± SEM) of *CCL26* and *DHRS3* expression in MDA-MB-231 cells shown. *RPL39* mRNA used as control in q-RT-PCR. *p < 0.05, **p < 0.01, ***p < 0.001. E entinostat, A all-trans retinoic acid, D doxorubicin, Veh vehicle, NS not significant, NA not available

Although immune deficient, athymic nude mice retain increased natural killer (NK) cell activity and tumoricidal macrophages [28]. Therefore, we investigated whether the increase in inflammatory genes in the tumor cells by ED and EAD treatment would also be followed by an increase in inflammation. The study pathologist’s quantification of inflammation in the tumor xenografts following the eight different treatments revealed that EAD treatment, followed by ED, resulted in significantly higher inflammatory scores (Fig. 5A and Additional file 13: Table S8). The inflammatory score consisted of primarily neutrophils and macrophages, some edema, and lesser numbers of lymphocytes.

These results in tumor xenografts showed that although EAD and ED equally regulated many proinflammatory genes (Fig. 4), EAD treatment in vivo showed a higher effect on the induction of inflammatory features in nude mice.

### EAD-specific inflammatory genes and regulation of *DHRS3*

As shown in Fig. 1a (and Additional file 2: Figure S1A) there is a high degree of overlap of the ED and EAD signatures and very few genes are unique to EAD’s triple drug effect in MDA-MB-231 cells. In order to identify genes that potentially contribute to the EAD’s effect on cell death and differentiation [20] and inflammation, we investigated differentially expressed genes between EAD and ED (Fig. 5B).

EAD treatment induced *ELF3* and *IL-1β* in comparison to ED (Fig. 5B). Previously, we have described* ELF3* as an EAD-induced gene with a role in differentiation ([20] and Fig. 5C). In addition, EAD upregulates *DHRS3*, *CCL26*, *TNFα*, *CD14*, *IL-1α*, and others (Fig. 5B and Additional file 14: Figure S5A). qRT-PCR validation showed that mRNA levels for the cytokine *CCL26* were induced in MDA-MB-231 following EAD treatment in comparison to ED and other treatments (Fig. 5Ca). Interestingly, the dehydrogenase/reductase member 3 (*DHRS3*, also known as retSDR1) which is involved in maintaining the cellular supply of retinol metabolites [29] was identified in the array as one the few genes induced by ATRA in comparison to vehicle. DHRS3 levels were higher in EAD-treated MDA-MB-231 (Fig. 5Cb) and SUM-159 (Additional file 14: Figure S5B) cells in comparison to ED and were also induced by all combinations of ATRA treatment, including the individual treatment. Similarly, ATRA and to a lesser extent entinostat increased DHRS3 protein levels in MDA-MB-231 cells (Fig. 5Cc and Additional file 14: Figure S5C). Treatment of MDA-MB-231 cells with EAD, in the presence of a low dose of doxorubicin (12.5 nM), was more effective than single agents and ED in induction of DHRS3 expression (Fig. 5Cc and Additional file 14: Figure S5C). Treatment of cells with a high dose of doxorubicin (200 nM) also increased DHRS3 expression and the combination with ATRA (AD) most effectively caused increased expression of this protein (Fig. 5Cc and Additional file 14: Figure S5C). *DHRS3* mRNA was also significantly higher in EAD-treated tumors in comparison to ED or vehicle (Fig. 5D). DHRS3 levels also tended to increase in the EA-treated tumors in comparison to vehicle (Fig. 5D). We also investigated *DHRS3* expression in normal breast, primary, and metastatic TNBC to correlate their levels with tumor progression. Metastatic TNBCs showed lower levels of *DHRS3* in comparison to primary tumors and normal breast (Fig. 5E). The role of *ELF3*, *CCL26*, and* DHRS3* in the induction of inflammation by EAD remains to be determined.

### ED-regulated genes are associated with better disease outcome in TNBC patients

**Fig. 6:**
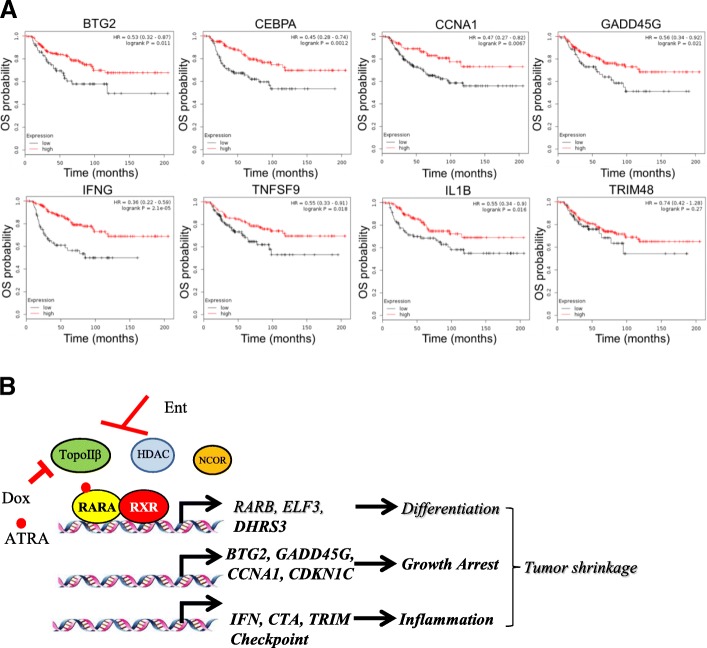
ED-induced genes correlate with better prognosis in TNBC patients. **a** Kaplan–Meier curves of overall survival (OS) showing correlation of ED-induced gene expression and prognosis in basal/TNBC patients, over a period of 12 years. **b** Scheme summarizing conclusions of this study on effect of EAD combination therapy in TNBCs. Ent entinostat, ATRA all-trans retinoic acid, Dox doxorubicin, HDAC histone deacetylase, RAR retinoic acid receptor, RXR retinoid X receptor, TopoIIβ topoisomerase II beta, NCOR nuclear receptor co-repressor

We next investigated whether there is a correlation of the epigenetic therapy-induced genes with survival in patients with TNBC. Higher expression of ED-induced genes such as *BTG2*, *CEBPA*, *CCNA1*, and *GADD45G* related to growth arrest, and *IFNG*, *TNFSF9*, *IL1B*, and *TRIM48* related to inflammatory response, significantly correlated with a better prognosis of patients with basal/TNBC. Longer overall survival (Fig. 6a, *n* = 241 patients), relapse-free survival (Additional file 15: Figure S6A, *n* = 614 patients), and metastases-free survival (Additional file 15: Figure S6B, *n* = 232 patients) were observed in TNBC patients with higher expression of ED-induced genes.

## Discussion

Previously, we have demonstrated that a combination of entinostat, ATRA, and doxorubicin (EAD) resulted in the greatest induction of cell death and cancer stem cell differentiation, and in consequence inhibition of xenografts of TNBC cells [20]. In this article, we show additional mechanisms likely to be involved in EAD-mediated decrease of tumor volume. Global gene expression analysis of TNBC cells following EAD and single treatments revealed that ED is a potent gene reprogramming therapy, and differentially regulated the expression of many genes more efficiently than single agents (Fig. 1).

We speculate that this ED effect on gene expression could be attributed to the inhibition of topoisomerase II-beta by both agents, as we have described previously in the case of *RAR-β* induction [20]. Pathway analysis showed that ED treatment regulated genes related to inflammation, growth arrest, and differentiation (Fig. 1). ED downregulated MYC and E2F targets and genes related to progression through the G2M checkpoint (Fig. 1). Recently, Topper et al. [30] showed that the combination of HDAC and DNA methyltransferase inhibitors decreased MYC-driven cell proliferation in lung cancer. In line with this finding related to the regulation of cell cycle arrest and death genes, we observed that entinostat treatment sensitized TNBC cells to doxorubicin-induced G2 cell cycle arrest (Fig. 2). Retinoic acid [3], doxorubicin [31], and HDACi [13] as single agents were also shown to induce cell arrest. Previously, we showed that ED and EAD increased apoptosis and EAD is the most effective to induce necrosis [20]. Collectively, these data showed that the increase in G2 cell arrest by ED and EAD (Fig. 2) led to cancer cell apoptosis and necrosis [20] and contributed to the decrease of tumor volume [20].

Among the inflammatory genes, ED increased the expression of interferon (IFN) genes, which correlated to the higher levels of infiltrated immune cells in patient breast tumors (Fig. 3). These findings suggest that the induction of IFN genes by the epigenetic treatment may favor immune cell recruitment to the tumor site. Type I IFN possesses the potent ability to activate several immune cell types [32, 33]. The presence of lymphocytic infiltration in early-stage breast cancer was associated with good prognosis and high response rates to neoadjuvant chemotherapy [34], especially in ER^−^/HER2^−^ tumors [35]. Interestingly, *CXCL10*, *IRF1*, and *STAT1*, shown to be expressed in breast cancers of patients who did not relapse [26], were also induced by EAD (Fig. 3). In addition to the regulation of interferon responsive genes, such as members of the tripartite motif (TRIM) family (Fig. 3), ED treatment also significantly induced the expression of members of the cancer/testis antigens (CTA) family in TNBC cells (Fig. 4). Epigenetic modifications including promoter hypomethylation and histone deacetylation have important roles in CTA gene activation [36]. CTAs are protein antigens normally expressed in a wide variety of malignant tumors but not in normal adult tissues, except for testis, and therefore have been viewed as attractive targets for cancer immunotherapy [37]. Similarly, 5-azacitidine regulates interferon signaling, antigen processing and presentation, cytokines/chemokines, and CTA genes [30, 38, 39]. In addition, we showed that several inflammatory genes related to immune cell activation and migration, including immune checkpoints, are also epigenetically regulated and induced by entinostat and further potentiated by the ED combination (Fig. 4). In fact, HDACi have been shown to enhance immunogenicity of cancer cells. Several groups have reported the upregulation of natural killer (NK) cell-activating ligands, MHC class I and II molecules, components of the machinery for antigen presentation, and costimulatory molecules on the surface of cancer cells exposed to HDACi [40–42]. Entinostat potentiates the effect of immune checkpoint-blocking antibodies, and the combination decreased regulatory T cells (Tregs) and myeloid derived suppressor cells (MDSC) in mice [43], and in patients [44]. Treatment with epigenetic therapy prevented exhaustion of CD8^+^ T cells and increased their expansion after immune-checkpoint blockade [45]. Chemotherapy was also shown to augment tumor immunity [46]. Previously we observed that EAD induced almost two times higher levels of necrosis compared to ED in vitro, and in tumor xenografts [20]. The increase in the number of dying cells in tumors of EAD-treated mice [1] may release damage-associated molecular patterns, release antigens, and initiate an immune response [47]. We found that entinostat in combination with doxorubicin (ED) most effectively upregulated interferon response, tumor antigens, cytokines, costimulatory molecules, and other inflammatory genes (Figs. 1, 3, and 4), and therefore may improve immunotherapies. Although we observed a similar effect of ED and EAD on the regulation of inflammatory genes, in-vivo EAD treatment was the most effective to increase the recruitment of immune cells and edema at the tumor site of nude mice (Fig. 5), the least immune-deficient mouse model [28].

ATRA is critical in maintaining immune homeostasis [4, 48] to differentiate myeloid-derived suppressor cells (MDSCs) into dendritic cells (DCs) and to improve their immunostimulatory capacity [49, 50]. Treatment of renal cell carcinoma patients with ATRA substantially decreased the presence of MDSCs in peripheral blood [51]. A recent study demonstrated that in lung cancer patients, p53 vaccine-generated immune responses were improved if patients received a short course of ATRA [52]. Some of the genes induced by EAD in comparison to ED, such as *ELF3* [53, 54], *DHRS3*, *IL-1β*, *CCL26 *[55], *TNF-α*, *CD14*, and *IL-1α *(Fig. 5 and Additional file 12: Figure S4), play a role in inflammation. *DHRS3* is involved in maintaining the cellular supply of retinol metabolites [29] and was described to be induced by the retinoid X receptor (RXR) rexinoid ligand bexarotene in MMTV-erbB2 mice [56]. We also observed a correlation of the epigenetic therapy-induced genes, related to growth arrest and inflammation, with survival in patients with TNBC. Collectively, these data suggested that ED and EAD treatment likely potentiates tumor immune surveillance.

## Conclusions

Some of the mechanisms involved in the EAD-induced decrease in tumor volume (Fig. 6b) include an increase in differentiation and cell death [20], due to an increase in cell cycle arrest. Entinostat potentiated doxorubicin-mediated cell death and the combination induced inflammatory signatures. The ED-induced immunomodulation may improve immunotherapy. Addition of ATRA to ED may potentiate inflammation and contribute to TNBC regression.

## Additional files

Additional file 1: Additional methods. (DOCX 45 kb)

Additional file 2: **Figure S1.** Gene expression array profile of TNBC cells identified entinostat and doxorubicin (ED) as a gene reprogramming combination. (**A**) Principal component analysis (PCA) 3D projection of gene expression data obtained from microarray analysis onto first three principal components. Each ball represents a different sample; different treatments indicated. 1 and 2 are duplicates from different batches. (**B**) Venn diagram showing number of genes common (intersection) and unique to each indicated treatment combination. Each treatment gene signature derived following normalization with genes present in MDA-MB-231 cells after vehicle (DMSO) treatment. Ingenuity® Pathway Analysis (IPA) generated network of (**C**) inflammatory and (**D**) cellular movement signaling upregulated in MDA-MB-231 cells treated with ED combination compared to single treatments. Color indicates genes upregulated (red) and downregulated (green) in MDA-MB-231 cells by ED treatment. Open and closed edges indicate genes with direct and indirect relationships respectively. E entinostat, A all-trans retinoic acid, D doxorubicin (different combinations). (PPTX 579 kb)

Additional file 3: **Table S1.** ED genes in comparison to entinostat and doxorubicin treatments. (DOCX 41 kb)

Additional file 4: **Table S2.** Genes differentially expressed by ED treatment and validated by qRT-PCR. (DOCX 14 kb)

Additional file 5: **Table S3.** Ingenuity® Pathway Analysis of ED genes (DOCX 13 kb)

Additional file 6: **Table S4.** Gene set analysis on ED genes. (DOCX 12 kb)

Additional file 7: **Figure S2.** ED and EAD induce cell growth arrest. (**A**) qRT-PCR of cyclin D1 (*CCND1*) and cyclin-dependent kinase 6 (*CDK6*) (identified by array analysis) in MDA-MB-231 cells treated with entinostat (2.5 μM), ATRA (1 μM), and doxorubicin (0.2 μM) singly, and combinations, for 48 h. (**B**) ImageJ quantification of cyclin D1 (left) and cyclin A (right) protein expression in MDA-MB-231 cells treated with entinostat (2.5 μM), ATRA (1 μM), and doxorubicin (0.2 μM) singly, and combinations, for 48 h. *Compared to entinostat in qRT-PCR: *p < 0.05, **p < 0.01. *t* test used to compare mean level of mRNA expression (± SEM), after *RPL39* normalization. (PPTX 75 kb)

Additional file 8: **Table S5.** ED and EAD induce growth arrest in HCC1937 TNBC cells. (DOCX 15 kb)

Additional file 9: **Table S6.** IFN-α genes induced by ED in MDA-MB-231 cells and correlated with immune infiltration. (DOCX 14 kb)

Additional file 10: **Figure S3.** ED induces interferon gamma genes associated with an increase in tumor lymphocytes. (**A**) Hierarchical supervised clustering of expression of interferon-gamma (IFN-G) genes against signatures of MDA-MB-231 cells following treatments. (**B**) qRT-PCR of (a) *IFN-G* in MDA-MB-231 and (b) CXCL10 and TRIM48 in SUM-159 cells treated with EAD singly and in combinations (doxorubicin 12.5 and 200 nM). (**C)** Unsupervised hierarchical cluster analysis of tumor-infiltrating lymphocyte genes [57], used in Fig. 3C to classify immune infiltration (low, medium, and high) in TCGA RNA-seq breast cancer patient dataset [58]. (**D**) Hierarchical supervised clustering of expression of IFN-γ genes against TCGA RNA-seq breast cancer patient dataset. Bars above identify different tumor subtypes (PAM50) and inflammatory cell content (immune, low–high) identified in (C). (**E**) One-way ANOVA showed significant difference across one or more groups (#1 low, #2 medium, #3 high immune cells) and post-hoc pairwise Student *t* test revealed statistically significant differences across all groups (*p* < 0.05). (**F**) IFN-γ score correlation with immune infiltration. *p < 0.05, **p < 0.01, ***p < 0.001. (PPTX 538 kb)

Additional file 11: **Table S7.** IFN-γ genes induced by ED in MDA-MB-231 cells and correlated with immune infiltration. (DOCX 15 kb)

Additional file 12: **Figure S4.** ED regulates expression of inflammatory genes. (**A**) qRT-PCR of SPANXN1 in SUM-159 cells treated as described in text (doxorubicin 200 nM). *t* test used to compare mean level of mRNA expression (± SEM) after *RPL39 *normalization. **p < 0.01, ***p < 0.001. (**B**) ImageJ quantification of GITRL, IL13RA2, and housekeeping β-actin protein in MDA-MB-231 cells treated as described (doxorubicin 12.5 and 200 nM). (PPTX 74 kb)

Additional file 13: **Table S8.** Inflammation score significance in mouse xenografts. (DOCX 14 kb)

Additional file 14: **Figure S5.** genesEAD regulates inflammatory genes. qRT-PCR of *TNF-α* (a) and *CD14* and *IL1a* (b) in MDA-MB-231 cells (A) and *DHRS3* in SUM-159 cells (B) treated as described in text (doxorubicin 12.5 and 200 nM). *t* test used to compare mean level of mRNA expression (± SEM) after *RPL39* normalization. *p < 0.05, **p < 0.01. (C) ImageJ quantification of DHRS3 and housekeeping β-actin proteins in MDA-MB-231 cells treated as described. (PPTX 105 kb)

Additional file 15: **Figure S6.** ED-induced genes correlate with a better prognosis in TNBC patients. Kaplan–Meier curves of relapsefree survival (RFS) (A) and metastases-free survival (DMFS) (B) showing correlation of ED-induced gene expression and prognosis in basal/TNBC patients, over a period of 12 years. (PPTX 378 kb)

## Abbreviations

ATRA: All-trans retinoic acid
CTA: Cancer testes antigens
DHRS3: Dehydrogenase/reductase member 3
GSEA: Gene set enrichment analysis
HDACi: Histone deacetylase inhibitors
IFN: Interferon
TCGA: The Cancer Genome Atlas
TNBC: Triple-negative breast cancer
TRIM: Tripartite motif

## References

[1] Ginestier C, Wicinski J, Cervera N, Monville F, Finetti P, Bertucci F, Wicha MS, Birnbaum D, Charafe-Jauffret E (2009). Retinoid signaling regulates breast cancer stem cell differentiation. Cell Cycle.

[2] Bhat-Nakshatri P, Goswami CP, Badve S, Sledge GW, Nakshatri H (2013). Identification of FDA-approved drugs targeting breast cancer stem cells along with biomarkers of sensitivity. Sci Rep.

[3] Seewaldt VL, Johnson BS, Parker MB, Collins SJ, Swisshelm K (1995). Expression of retinoic acid receptor beta mediates retinoic acid-induced growth arrest and apoptosis in breast cancer cells. Cell Growth Differ.

[4] Hall JA, Grainger JR, Spencer SP, Belkaid Y (2011). The role of retinoic acid in tolerance and immunity. Immunity.

[5] Connolly RM, Nguyen NK, Sukumar S (2013). Molecular pathways: current role and future directions of the retinoic acid pathway in cancer prevention and treatment. Clin Cancer Res.

[6] Anso E, Mullen AR, Felsher DW, Mates JM, Deberardinis RJ, Chandel NS (2013). Metabolic changes in cancer cells upon suppression of MYC. Cancer Metab.

[7] Tang XH, Gudas LJ (2011). Retinoids, retinoic acid receptors, and cancer. Annu Rev Pathol.

[8] Sirchia SM, Ferguson AT, Sironi E, Subramanyan S, Orlandi R, Sukumar S, Sacchi N (2000). Evidence of epigenetic changes affecting the chromatin state of the retinoic acid receptor beta2 promoter in breast cancer cells. Oncogene.

[9] Nacht M, Ferguson AT, Zhang W, Petroziello JM, Cook BP, Gao YH, Maguire S, Riley D, Coppola G, Landes GM (1999). Combining serial analysis of gene expression and array technologies to identify genes differentially expressed in breast cancer. Cancer Res.

[10] Li Yixuan, Seto Edward (2016). HDACs and HDAC Inhibitors in Cancer Development and Therapy. Cold Spring Harbor Perspectives in Medicine.

[11] Connolly Roisin M, Rudek Michelle A, Piekarz Richard (2017). Entinostat: a promising treatment option for patients with advanced breast cancer. Future Oncology.

[12] Vigushin DM, Ali S, Pace PE, Mirsaidi N, Ito K, Adcock I, Coombes RC (2001). Trichostatin A is a histone deacetylase inhibitor with potent antitumor activity against breast cancer in vivo. Clin Cancer Res.

[13] Huang X, Gao L, Wang S, Lee CK, Ordentlich P, Liu B (2009). HDAC inhibitor SNDX-275 induces apoptosis in erbB2-overexpressing breast cancer cells via down-regulation of erbB3 expression. Cancer Res.

[14] Ray A, Alalem M, Ray BK (2013). Loss of epigenetic Kruppel-like factor 4 histone deacetylase (KLF-4-HDAC)-mediated transcriptional suppression is crucial in increasing vascular endothelial growth factor (VEGF) expression in breast cancer. J Biol Chem.

[15] Kaluza D, Kroll J, Gesierich S, Yao TP, Boon RA, Hergenreider E, Tjwa M, Rossig L, Seto E, Augustin HG (2011). Class IIb HDAC6 regulates endothelial cell migration and angiogenesis by deacetylation of cortactin. EMBO J.

[16] Salvador MA, Wicinski J, Cabaud O, Toiron Y, Finetti P, Josselin E, Lelievre H, Kraus-Berthier L, Depil S, Bertucci F (2013). The histone deacetylase inhibitor abexinostat induces cancer stem cells differentiation in breast cancer with low Xist expression. Clin Cancer Res.

[17] West AC, Smyth MJ, Johnstone RW (2014). The anticancer effects of HDAC inhibitors require the immune system. Oncoimmunology.

[18] Munster PN, Marchion D, Thomas S, Egorin M, Minton S, Springett G, Lee JH, Simon G, Chiappori A, Sullivan D (2009). Phase I trial of vorinostat and doxorubicin in solid tumours: histone deacetylase 2 expression as a predictive marker. Br J Cancer.

[19] Pili R, Salumbides B, Zhao M, Altiok S, Qian D, Zwiebel J, Carducci MA, Rudek MA (2012). Phase I study of the histone deacetylase inhibitor entinostat in combination with 13-cis retinoic acid in patients with solid tumours. Br J Cancer.

[20] Merino VF, Nguyen N, Jin K, Sadik H, Cho S, Korangath P, Han L, Foster YM, Zhou XC, Zhang Z (2016). Combined treatment with epigenetic, differentiating, and chemotherapeutic agents cooperatively targets tumor-initiating cells in triple-negative breast cancer. Cancer Res.

[21] Gyorffy B, Lanczky A, Eklund AC, Denkert C, Budczies J, Li Q, Szallasi Z (2010). An online survival analysis tool to rapidly assess the effect of 22,277 genes on breast cancer prognosis using microarray data of 1,809 patients. Breast Cancer Res Treat.

[22] Gyorffy B, Surowiak P, Budczies J, Lanczky A (2013). Online survival analysis software to assess the prognostic value of biomarkers using transcriptomic data in non-small-cell lung cancer. PLoS One.

[23] Hanzelmann S, Castelo R, Guinney J (2013). GSVA: gene set variation analysis for microarray and RNA-seq data. BMC Bioinformatics.

[24] Jin G, Bausch D, Knightly T, Liu Z, Li Y, Liu B, Lu J, Chong W, Velmahos GC, Alam HB (2011). Histone deacetylase inhibitors enhance endothelial cell sprouting angiogenesis in vitro. Surgery.

[25] Alao JP, Lam EW, Ali S, Buluwela L, Bordogna W, Lockey P, Varshochi R, Stavropoulou AV, Coombes RC, Vigushin DM (2004). Histone deacetylase inhibitor trichostatin A represses estrogen receptor alpha-dependent transcription and promotes proteasomal degradation of cyclin D1 in human breast carcinoma cell lines. Clin Cancer Res.

[26] Ascierto ML, Kmieciak M, Idowu MO, Manjili R, Zhao Y, Grimes M, Dumur C, Wang E, Ramakrishnan V, Wang XY (2012). A signature of immune function genes associated with recurrence-free survival in breast cancer patients. Breast Cancer Res Treat.

[27] Yao J, Caballero OL, Yung WK, Weinstein JN, Riggins GJ, Strausberg RL, Zhao Q (2014). Tumor subtype-specific cancer-testis antigens as potential biomarkers and immunotherapeutic targets for cancers. Cancer Immunol Res.

[28] Zhou Q, Facciponte J, Jin M, Shen Q, Lin Q (2014). Humanized NOD-SCID IL2rg−/− mice as a preclinical model for cancer research and its potential use for individualized cancer therapies. Cancer Lett.

[29] Haeseleer F, Huang J, Lebioda L, Saari JC, Palczewski K (1998). Molecular characterization of a novel short-chain dehydrogenase/reductase that reduces all-trans-retinal. J Biol Chem.

[30] Topper MJ, Vaz M, Chiappinelli KB, DeStefano Shields CE, Niknafs N, Yen RC, Wenzel A, Hicks J, Ballew M, Stone M (2017). Epigenetic therapy ties MYC depletion to reversing immune evasion and treating lung cancer. Cell.

[31] Bar-On O, Shapira M, Hershko DD (2007). Differential effects of doxorubicin treatment on cell cycle arrest and Skp2 expression in breast cancer cells. Anti-Cancer Drugs.

[32] Zitvogel L, Galluzzi L, Kepp O, Smyth MJ, Kroemer G (2015). Type I interferons in anticancer immunity. Nat Rev Immunol.

[33] Minn AJ, Wherry EJ (2016). Combination cancer therapies with immune checkpoint blockade: convergence on interferon signaling. Cell.

[34] Denkert C, Loibl S, Noske A, Roller M, Muller BM, Komor M, Budczies J, Darb-Esfahani S, Kronenwett R, Hanusch C (2010). Tumor-associated lymphocytes as an independent predictor of response to neoadjuvant chemotherapy in breast cancer. J Clin Oncol.

[35] Loi S, Sirtaine N, Piette F, Salgado R, Viale G, Van Eenoo F, Rouas G, Francis P, Crown JP, Hitre E (2013). Prognostic and predictive value of tumor-infiltrating lymphocytes in a phase III randomized adjuvant breast cancer trial in node-positive breast cancer comparing the addition of docetaxel to doxorubicin with doxorubicin-based chemotherapy: BIG 02-98. J Clin Oncol.

[36] De Smet C, Lurquin C, Lethe B, Martelange V, Boon T (1999). DNA methylation is the primary silencing mechanism for a set of germ line- and tumor-specific genes with a CpG-rich promoter. Mol Cell Biol.

[37] Whitehurst AW (2014). Cause and consequence of cancer/testis antigen activation in cancer. Annu Rev Pharmacol Toxicol.

[38] Roulois D, Loo Yau H, Singhania R, Wang Y, Danesh A, Shen SY, Han H, Liang G, Jones PA, Pugh TJ (2015). DNA-demethylating agents target colorectal cancer cells by inducing viral mimicry by endogenous transcripts. Cell.

[39] Li H, Chiappinelli KB, Guzzetta AA, Easwaran H, Yen RW, Vatapalli R, Topper MJ, Luo J, Connolly RM, Azad NS (2014). Immune regulation by low doses of the DNA methyltransferase inhibitor 5-azacitidine in common human epithelial cancers. Oncotarget.

[40] Khan AN, Magner WJ, Tomasi TB (2007). An epigenetic vaccine model active in the prevention and treatment of melanoma. J Transl Med.

[41] Magner WJ, Kazim AL, Stewart C, Romano MA, Catalano G, Grande C, Keiser N, Santaniello F, Tomasi TB (2000). Activation of MHC class I, II, and CD40 gene expression by histone deacetylase inhibitors. J Immunol.

[42] Skov S, Pedersen MT, Andresen L, Straten PT, Woetmann A, Odum N (2005). Cancer cells become susceptible to natural killer cell killing after exposure to histone deacetylase inhibitors due to glycogen synthase kinase-3-dependent expression of MHC class I-related chain A and B. Cancer Res.

[43] Kim K, Skora AD, Li Z, Liu Q, Tam AJ, Blosser RL, Diaz LA, Papadopoulos N, Kinzler KW, Vogelstein B (2014). Eradication of metastatic mouse cancers resistant to immune checkpoint blockade by suppression of myeloid-derived cells. Proc Natl Acad Sci U S A.

[44] Tomita Y, Lee MJ, Lee S, Tomita S, Chumsri S, Cruickshank S, Ordentlich P, Trepel JB (2016). The interplay of epigenetic therapy and immunity in locally recurrent or metastatic estrogen receptor-positive breast cancer: correlative analysis of ENCORE 301, a randomized, placebo-controlled phase II trial of exemestane with or without entinostat. Oncoimmunology.

[45] Ghoneim HE, Fan Y, Moustaki A, Abdelsamed HA, Dash P, Dogra P, Carter R, Awad W, Neale G, Thomas PG (2017). De novo epigenetic programs inhibit PD-1 blockade-mediated T cell rejuvenation. Cell.

[46] Emens LA (2008). Chemotherapy and tumor immunity: an unexpected collaboration. Front Biosci.

[47] Kono K, Mimura K, Kiessling R (2013). Immunogenic tumor cell death induced by chemoradiotherapy: molecular mechanisms and a clinical translation. Cell Death Dis.

[48] Raverdeau M, Mills KH (2014). Modulation of T cell and innate immune responses by retinoic Acid. J Immunol.

[49] Kusmartsev S, Cheng F, Yu B, Nefedova Y, Sotomayor E, Lush R, Gabrilovich D (2003). All-trans-retinoic acid eliminates immature myeloid cells from tumor-bearing mice and improves the effect of vaccination. Cancer Res.

[50] Kusmartsev S, Su Z, Heiser A, Dannull J, Eruslanov E, Kubler H, Yancey D, Dahm P, Vieweg J (2008). Reversal of myeloid cell-mediated immunosuppression in patients with metastatic renal cell carcinoma. Clin Cancer Res.

[51] Mirza N, Fishman M, Fricke I, Dunn M, Neuger AM, Frost TJ, Lush RM, Antonia S, Gabrilovich DI (2006). All-trans-retinoic acid improves differentiation of myeloid cells and immune response in cancer patients. Cancer Res.

[52] Iclozan C, Antonia S, Chiappori A, Chen DT, Gabrilovich D (2013). Therapeutic regulation of myeloid-derived suppressor cells and immune response to cancer vaccine in patients with extensive stage small cell lung cancer. Cancer Immunol Immunother.

[53] Oliver JR, Kushwah R, Hu J (2012). Multiple roles of the epithelium-specific ETS transcription factor, ESE-1, in development and disease. Lab Invest.

[54] Kushwah R, Oliver JR, Wu J, Chang Z, Hu J (2011). Elf3 regulates allergic airway inflammation by controlling dendritic cell-driven T cell differentiation. J Immunol.

[55] Kitaura M, Suzuki N, Imai T, Takagi S, Suzuki R, Nakajima T, Hirai K, Nomiyama H, Yoshie O (1999). Molecular cloning of a novel human CC chemokine (Eotaxin-3) that is a functional ligand of CC chemokine receptor 3. J Biol Chem.

[56] Li Y, Zhang Y, Hill J, Kim HT, Shen Q, Bissonnette RP, Lamph WW, Brown PH (2008). The rexinoid, bexarotene, prevents the development of premalignant lesions in MMTV-erbB2 mice. Br J Cancer.

[57] Karn T, Pusztai L, Ruckhaberle E, Liedtke C, Muller V, Schmidt M, Metzler D, Wang J, Coombes KR, Gatje R (2012). Melanoma antigen family A identified by the bimodality index defines a subset of triple negative breast cancers as candidates for immune response augmentation. Eur J Cancer.

[58] Cancer Genome Atlas Network, Comprehensive molecular portraits of human breast tumours (2012). Nature.

