# Supplementary material for: Induction of cell cycle arrest and inflammatory genes by combined treatment with epigenetic, differentiating, and chemotherapeutic agents in triple-negative breast cancer
Source: Breast Cancer Res. 2018 Nov 28;20:145. doi: 10.1186/s13058-018-1068-x (PMC6263070; doi:10.1186/s13058-018-1068-x)
Supplement: Supplementary file 1 — Additional methods. (DOCX 45 kb) [file 13058_2018_1068_MOESM1_ESM.docx]

**Additional Methods**

**Transcriptome array.** RNA was hybridized onto Illumina’s HumanHT-12v4 BeadChip (Illumina, San Diego) using manufacturer recommended protocols. These chips provide comprehensive coverage of >47,000 transcripts, including all current NCBI curated genes. After pre-processing using Illumina GenomeStudio software, quantile normalization was applied to log transformed intensities for future analyses. Statistical analyses were performed using the R statistical software with standard R packages from the Bioconductor Bioinformatics Software Project, and customized programs when necessary.

**PCA analysis and plot.** Principle component analysis (PCA) was performed and visualized using Partek Genomics Suite (PGS) on gene expression profiles of MDA-MB-231 cells following the eight different treatments. Probe intensities were treated as feature variables where samples were projected to the principle components. PGS plots the PCA results by projecting each sample to the first three principal components. The distances projected on the three axes represent the differences between samples and samples with similar gene expression profiles were closer together.

**Unsupervised clustering.** Unsupervised clustering was performed by identifying the five hundred most variable probes by standard deviation across all MDA-MB-231 treatment groups. Hierarchical clustering using Ward’s distance was performed on this subset of genes.

**Batch correction.** Batch correction was performed using a linear regression model for the MDA-MB-231 gene expression profiles where the batch status was fitted as a variable using the R package, linear models of microarray data (limma). The residuals of that coefficient were extracted from the Bayesian model and summed with the intercept to obtain the final batch-corrected dataset for input into gene set analysis.

**Differential expression analysis.** Differential expression analysis across MDA-MB-231 treatment groups was performed using limma with batch being a covariate (see batch correction) in the model matrix. These comparisons were performed individually for each treatment group against DMSO control. Probes are considered differentially expressed when the fold-change is equal or greater than two and the false discovery rate (FDR) < 0.05. The ED signature was derived by subtracting probe changes in ED combination treated cells against changes in E single + D single treatment cells in the limma model. Unless otherwise stated, the log2 fold-change and FDR from limma were plotted as a volcano plot, where the y-axis represents –log_10_ FDR values with higher values being more significant and x-axis represents fold change. Venn diagrams using the gplots package were used to visualize overlapping sets of genes across treatment groups.

**Gene Set Variation Analysis** (**GSVA).** GSVA scores for the MDA-MB-231 gene expression profiles of all 8 treatment groups were estimated using GSVA with default parameters against the MSigDB Hallmark gene sets. 100-fold bootstrapping was performed to identify statistically significant GSVA scores (p <= 0.05). ANOVA was performed to identify gene sets with statistically significant difference across the treatment groups.

**Functional and pathway analysis by Ingenuity® Pathway Analysis (IPA).** The ED gene expression signature (ED_double_ – (E_single_+D_single_)) was uploaded into the Ingenuity Pathway Analysis (IPA) software (Ingenuity Systems, Redwood City, CA) and probes were defined as significant when the FDR < 0.05 and had a minimum 2-fold change. Networks of these genes were algorithmically generated based on their connectivity and assigned a score, which is a numerical value used to rank networks according to how relevant they are to the genes. The significance of the association between the data set and the canonical pathway was determined based on two parameters: (1) A ratio of the number of genes from the data set that map to the pathway divided by the total number of genes that map to the canonical pathway and (2) a P value calculated using Fischer's exact test determining the probability that the association between the genes in the data set and the canonical pathway is greater than chance alone.

**Gene set analysis** **(GSEA).** Gene set analysis against Molecular Signatures Database (MSigDB) Hallmark gene sets was performed using the wilcoxGST function provided by the limma package. It is a rank-based GSEA approach, with p-value corrections performed using the Benjamini-Hochberg false discovery rate. The gene universe (background) for the test is set an intersection of all genes represented in MSigDB and TCGA RNA-seq data. The Hallmark gene sets were used as they were curated to represent major biological processes.

**RNA extraction, cDNA and Real-time Quantitative PCR.** Cells were seeded in 6 well plates and harvested at equal density and treated with TRIzol (Life Technologies). Mouse tumors were excised, snap frozen, pulverized and homogenized in TRIzol according to manufacturer’s recommendations (Life Technologies). RNA was quantified using a Nanodrop spectrophotometer (Thermo Scientific). 0.5- 1 ug of RNA was reversed transcribed using SuperScript® III Reverse Transcriptase and oligo (dT) (Life Technologies). Primers were designed using Primer 3 ([http://primer3.ut.ee](http://bioinfo.ut.ee/primer3/)) and Ensembl Genome Browser to span an intron. Real-time quantitative PCR was conducted using the Maxima SYBR Green/ROX Master Mix (Fermentas), per manufacturer protocol, using the Applied Biosystem 7500 Real-Time PCR System for 40 cycles. Relative expression was calculated by the ΔΔCt method, with RPL39 expression used for normalization. Primers sequences are provided upon request.

**Cell Cycle quantification by Flow Cytometry.** For cell cycle determination, cells were permeabilized overnight with cold 70% ethanol at −20°C. Cells were then pelleted and ressuspended in an isotonic buffered PI-staining solution containing RNase A (0.1 mg/ml, Qiagen, #19101) and Propidium Iodide (20 μg/mL, Sigma, #P-4864). Samples were run on the BD FACSCalibur system (Becton Dickinson), and data analyzed using FlowJo software.

**Western Blot Analysis**. Cells were seeded in 6 well plates and treated with the appropriate drug. At the indicated time, they were lysed with RIPA buffer containing protesase and phosphatase inhibitors (Roche, #1183615300 and #14906845001, respectively). 20 μg of extracted protein were vertically electrophoresed on 4-12% Bis-Tris NuPage Novex Gel in MOPS SDS running buffer (Invitrogen), then transferred to Hybond C Extra membrane (GE Healthcare). Membranes were stained with Ponceau stain to confirm protein transfer, then blocked with 5% powdered milk in PBS with 0.2% Tween-20 (PBST) for one hour. Membranes were probed with primary antibody in 5% milk/PBST at 4^o^C overnight, rinsed with PBST, then probed with secondary antibody (GE Healthcare) at 1:2000 dilution in 5% milk/PBST for 1h. After rinsing with PBST, membranes were treated with ECL Plus Detection Reagent (GE Healthcare) for 1 minute, and exposed to Hyblot CL autoradiography film to determine protein expression. Antibodies to Cyclin A (#574900), Cyclin D1 (#AHF0102) and GITRL (PA576208)from Thermo Scientific; GAPDH (#MAB374) and Beta-actin (A1978) from Millipore/ Sigma; DHRS3 (AB198005) from Abcam; IL13RA2 (sc-74160) from Santa Cruz Biotechnology. Quantitation was done using ImageJ software.

**Interferon, immune cells content, and PAM50 status.** Interferon (IFN) gene sets were obtained from the MSigDB Hallmark genesets. Since these gene sets consist of genes with positive enrichment, IFN scores were calculated as the mean expression of these set of genes. 10,000-fold permutation analysis where random sampling of N genes (where N = number of genes in each gene set) to generate a null distribution was performed to estimate the probability of observing a positive relationship between IFN scores and immune infiltration scores. Genes uniquely expressed by tumor infiltrating lymphocytes (TILs) were obtained from a study by Karn et al. [[1](#_ENREF_1)]. Gene level RNA-Seq Expectation Maximization (RSEM) values for the TIL gene set was obtained from the Cancer Genome Atlas (TCGA) BRCA RNA-seq2.0 dataset (https://tcga-data.nci.nih.gov/tcga/) [[2](#_ENREF_2)]. The RSEM values were log2 transformed and Z-scores (calculated as $\frac{x-\bar{x}}{\sigma}$) were performed on a gene level using the scale function in R Statistical Program. This was performed to standardize differences across genes so that equal weight is given to each gene. These Z-scores were used to cluster the samples into different groups of TIL infiltration using unsupervised hierarchical clustering and the optimal number of clusters was determined by maximizing the average silhouette width. The gene set and final samples used are shown in Fig. S3E. PAM50 status was predicted using the pam50.robust function from the genefu package ([http://www.pmgenomics.ca/bhklab/](https://mobile.johnshopkins.edu/owa/redir.aspx?C=1yN8hDbtuESmHMq7_WlU866PiUukGdIIqitmoLe7Sp1H1gygatdifUz7ByAdpR-NtGh26NnldNs.&URL=http%3a%2f%2fwww.pmgenomics.ca%2fbhklab%2f)) mapped by EntrezIDs.

**Histology: Scoring inflammation.** The primary tumor xenografts from the mice treated with the 8 different regimens were fixed in 10% buffered formalin, paraffin embedded, cut as 5 µm sections and stained with H&E. Histopathology of the H&E stained tissue sections was scored separately for severity and extent of inflammation, with the pathologist blinded to group of origin, using a scale of 0–3 as follows: No significant inflammation= 0; minimal to mild inflammatory infiltrates with edema= 1; moderate inflammatory infiltrates with edema= 2; marked inflammatory infiltrates with edema= 3. The inflammation consisted primarily of neutrophils and macrophages along with edema and lesser numbers of lymphocytes.

**Correlation of Gene Expression with Outcome.** We identified breast cancer samples in the Gene Expression Omnibus repository (http://www.ncbi.nlm.nih.gov/gds) using the GEO platform IDs "GPL96", "GPL570", "GPL571", "GPL6947" and "GPL4133" as well as the keywords "breast", "cancer", "chemotherapy" and "survival" as described previously [[3](#_ENREF_3)]. The search was restricted to datasets that included at least 30 patients. Altogether data from 5,935 breast cancer patients was downloaded and processed. Among these, 4,659 patients had survival information. In these patients, basal/triple negative breast cancer (TNBC) was defined as ER negative and HER2 negative using gene expression data (the probe for PGR is not reliable on the arrays). Patients in a large dataset (GSE25066, n= 507) received anthracycline treatment – these patients were analyzed separately so that the two investigated cohorts (basal and anthracycline-treated) do not overlap. The raw .CEL files were normalized using MAS5 in the R statistical environment (http://www.r-project.org) using the Affy Bioconductor library. Kaplan-Meier survival plot, and the hazard ratio with 95% confidence intervals and logrank P value were calculated and plotted in R using the Bioconductor package "survival" as described earlier [[4](#_ENREF_4)]. Statistical significance was set at p<0.01. Cox proportional hazard regression was performed to compare the association between gene expression, clinical variables and relapse-free survival using WinSTAT 2007 for Microsoft Excel (Robert K. Fitch Software, Germany).

**Statistical Analysis.** The cell line results were expressed as mean ± standard errors of mean (SEM). Two-tailed Student’s T-tests (95% confidence interval) were performed on pairwise combinations of data to determine statistical significance defined as *p < 0.05, **p< 0.01 and ***p< 0.001. qRT-PCR using tumor xenografts results were expressed using the median and two-tailed Mann Whitney Test. Statistical analyses were performed using GraphPad Prism version 5.0 (GraphPad Software, Inc.). Ordinal logistic regressions were used to estimate the likelihood of the inflammatory quantification from the tumor xenografts.

**References**

1. Karn T, Pusztai L, Ruckhaberle E, Liedtke C, Muller V, Schmidt M, Metzler D, Wang J, Coombes KR, Gatje R *et al*: **Melanoma antigen family A identified by the bimodality index defines a subset of triple negative breast cancers as candidates for immune response augmentation**. *Eur J Cancer* 2012, **48**(1):12-23.

2. **Comprehensive molecular portraits of human breast tumours**. *Nature* 2012, **490**(7418):61-70.

3. Gyorffy B, Lanczky A, Eklund AC, Denkert C, Budczies J, Li Q, Szallasi Z: **An online survival analysis tool to rapidly assess the effect of 22,277 genes on breast cancer prognosis using microarray data of 1,809 patients**. *Breast Cancer Res Treat* 2010, **123**(3):725-731.

4. Gyorffy B, Surowiak P, Budczies J, Lanczky A: **Online survival analysis software to assess the prognostic value of biomarkers using transcriptomic data in non-small-cell lung cancer**. *PLoS One* 2013, **8**(12):e82241.
