# Supplementary figures and images for: Induction of cell cycle arrest and inflammatory genes by combined treatment with epigenetic, differentiating, and chemotherapeutic agents in triple-negative breast cancer
Source: Breast Cancer Res. 2018 Nov 28;20:145. doi: 10.1186/s13058-018-1068-x (PMC6263070; doi:10.1186/s13058-018-1068-x)

## Slide 1
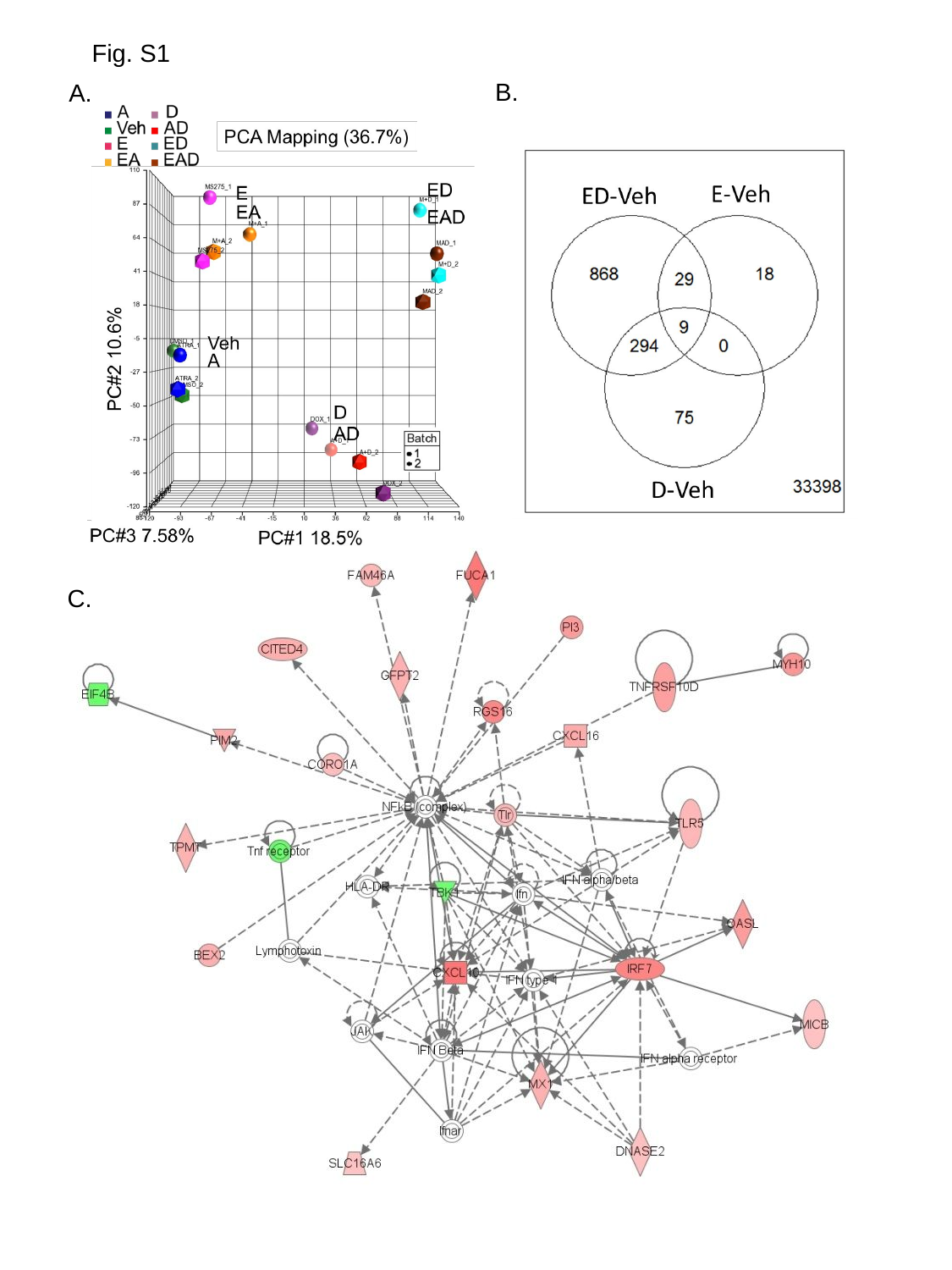

Fig. S1
B.
A.
C.

## Slide 2
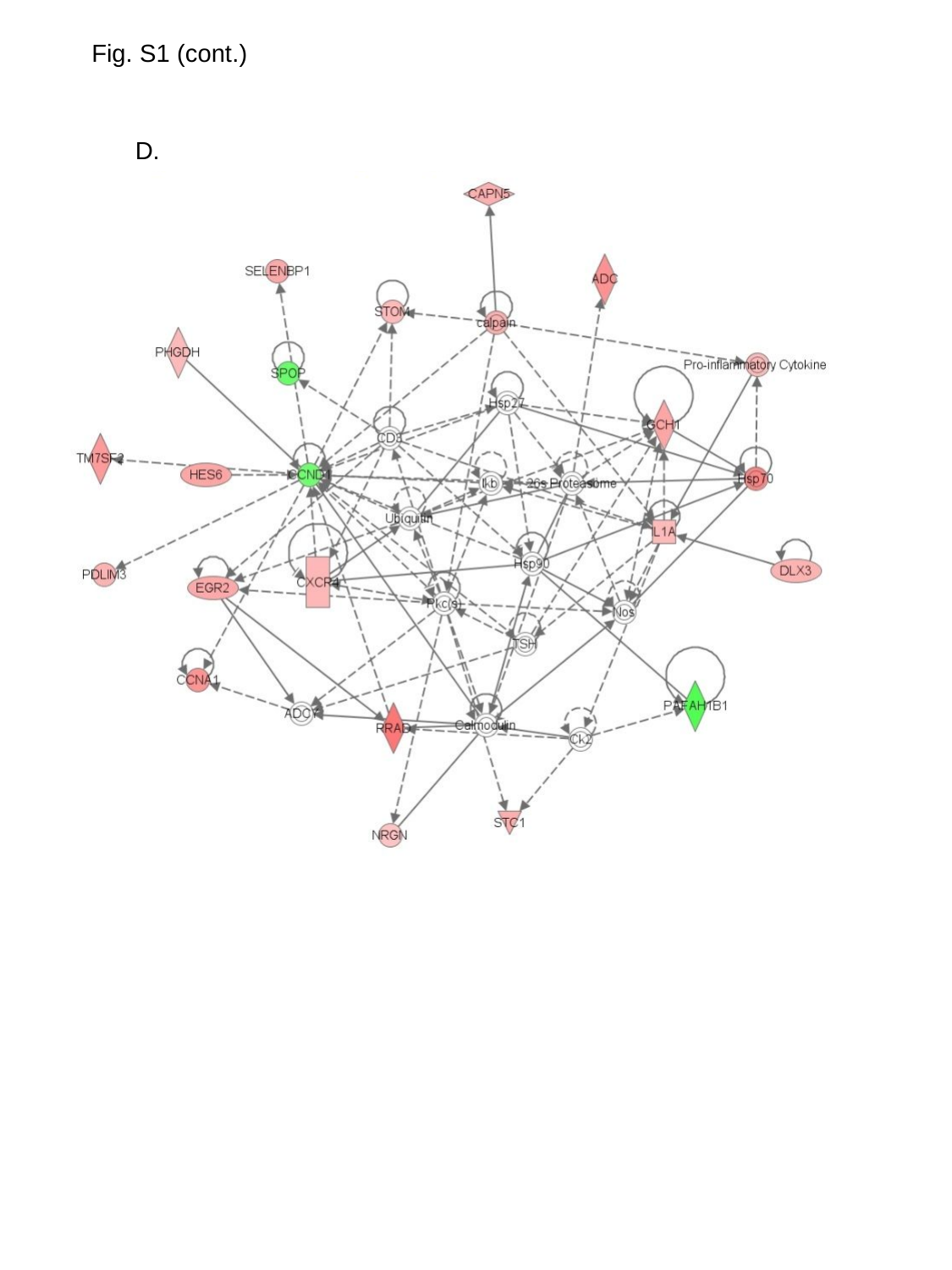

Fig. S1 (cont.)
D.

Supplement: Supplementary file 2 — Figure S1. Gene expression array profile of TNBC cells identified entinostat and doxorubicin (ED) as a gene reprogramming combination. (A) Principal component analysis (PCA) 3D projection of gene expression data obtained from microarray analysis onto first three principal components. Each ball represents a different sample; different treatments indicated. 1 and 2 are duplicates from different batches. (B) Venn diagram showing number of genes common (intersection) and unique to each indicated treatment combination. Each treatment gene signature derived following normalization with genes present in MDA-MB-231 cells after vehicle (DMSO) treatment. Ingenuity® Pathway Analysis (IPA) generated network of (C) inflammatory and (D) cellular movement signaling upregulated in MDA-MB-231 cells treated with ED combination compared to single treatments. Color indicates genes upregulated (red) and downregulated (green) in MDA-MB-231 cells by ED treatment. Open and closed edges indicate genes with direct and indirect relationships respectively. E entinostat, A all-trans retinoic acid, D doxorubicin (different combinations). (PPTX 579 kb) [file 13058_2018_1068_MOESM2_ESM.pptx]

## Slide 1
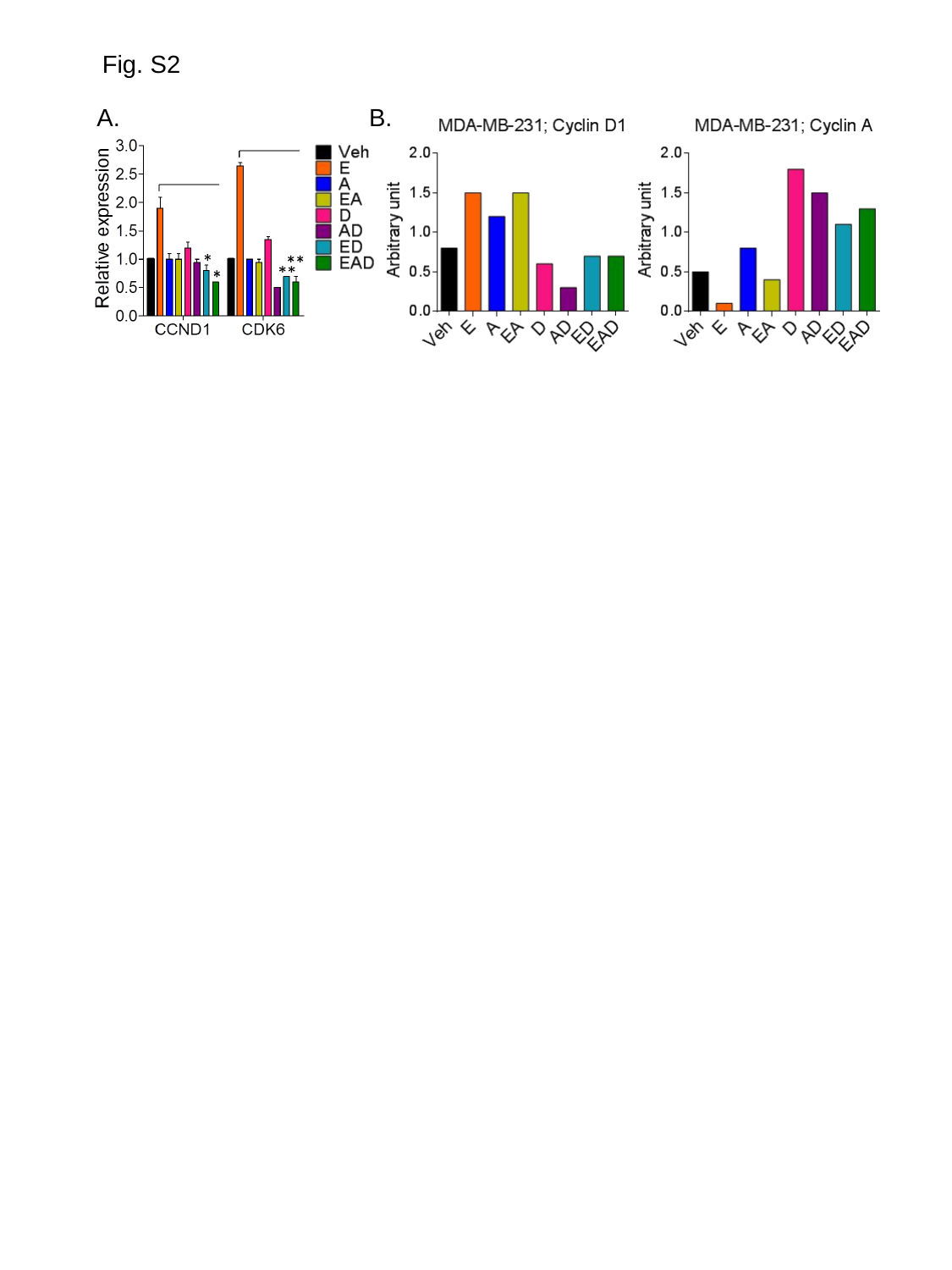

Fig. S2
B.
A.

Supplement: Supplementary file 7 — Figure S2. ED and EAD induce cell growth arrest. (A) qRT-PCR of cyclin D1 (CCND1) and cyclin-dependent kinase 6 (CDK6) (identified by array analysis) in MDA-MB-231 cells treated with entinostat (2.5 μM), ATRA (1 μM), and doxorubicin (0.2 μM) singly, and combinations, for 48 h. (B) ImageJ quantification of cyclin D1 (left) and cyclin A (right) protein expression in MDA-MB-231 cells treated with entinostat (2.5 μM), ATRA (1 μM), and doxorubicin (0.2 μM) singly, and combinations, for 48 h. *Compared to entinostat in qRT-PCR: *p < 0.05, **p < 0.01. t test used to compare mean level of mRNA expression (± SEM), after RPL39 normalization. (PPTX 75 kb) [file 13058_2018_1068_MOESM7_ESM.pptx]

## Slide 1
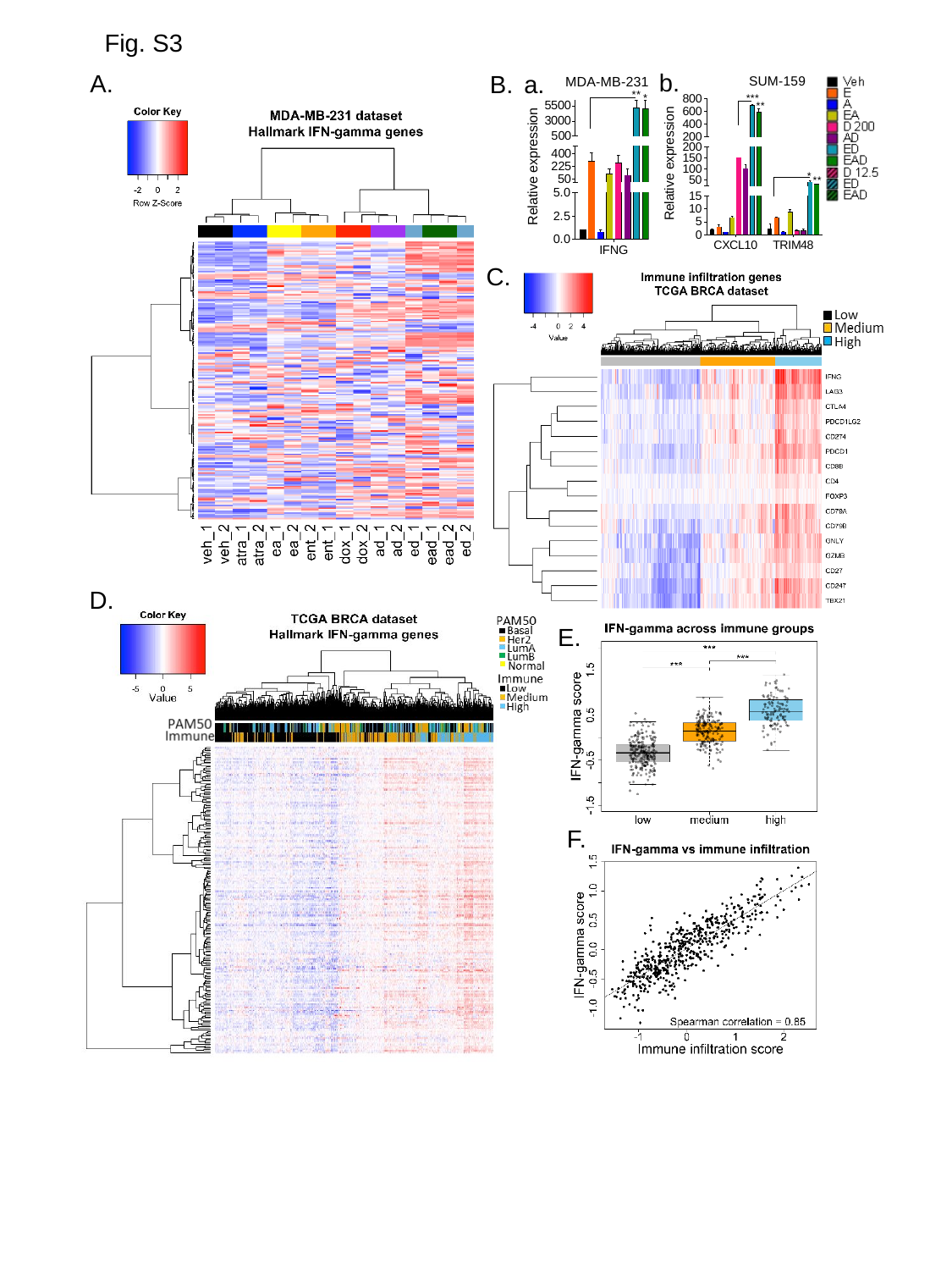

Fig. S3
b.
A.
a.
B.
C.
D.
E.
F.

Supplement: Supplementary file 10 — Figure S3. ED induces interferon gamma genes associated with an increase in tumor lymphocytes. (A) Hierarchical supervised clustering of expression of interferon-gamma (IFN-G) genes against signatures of MDA-MB-231 cells following treatments. (B) qRT-PCR of (a) IFN-G in MDA-MB-231 and (b) CXCL10 and TRIM48 in SUM-159 cells treated with EAD singly and in combinations (doxorubicin 12.5 and 200 nM). (C) Unsupervised hierarchical cluster analysis of tumor-infiltrating lymphocyte genes [57], used in Fig. 3C to classify immune infiltration (low, medium, and high) in TCGA RNA-seq breast cancer patient dataset [58]. (D) Hierarchical supervised clustering of expression of IFN-γ genes against TCGA RNA-seq breast cancer patient dataset. Bars above identify different tumor subtypes (PAM50) and inflammatory cell content (immune, low–high) identified in (C). (E) One-way ANOVA showed significant difference across one or more groups (#1 low, #2 medium, #3 high immune cells) and post-hoc pairwise Student t test revealed statistically significant differences across all groups (p < 0.05). (F) IFN-γ score correlation with immune infiltration. *p < 0.05, **p < 0.01, ***p < 0.001. (PPTX 538 kb) [file 13058_2018_1068_MOESM10_ESM.pptx]

## Slide 1
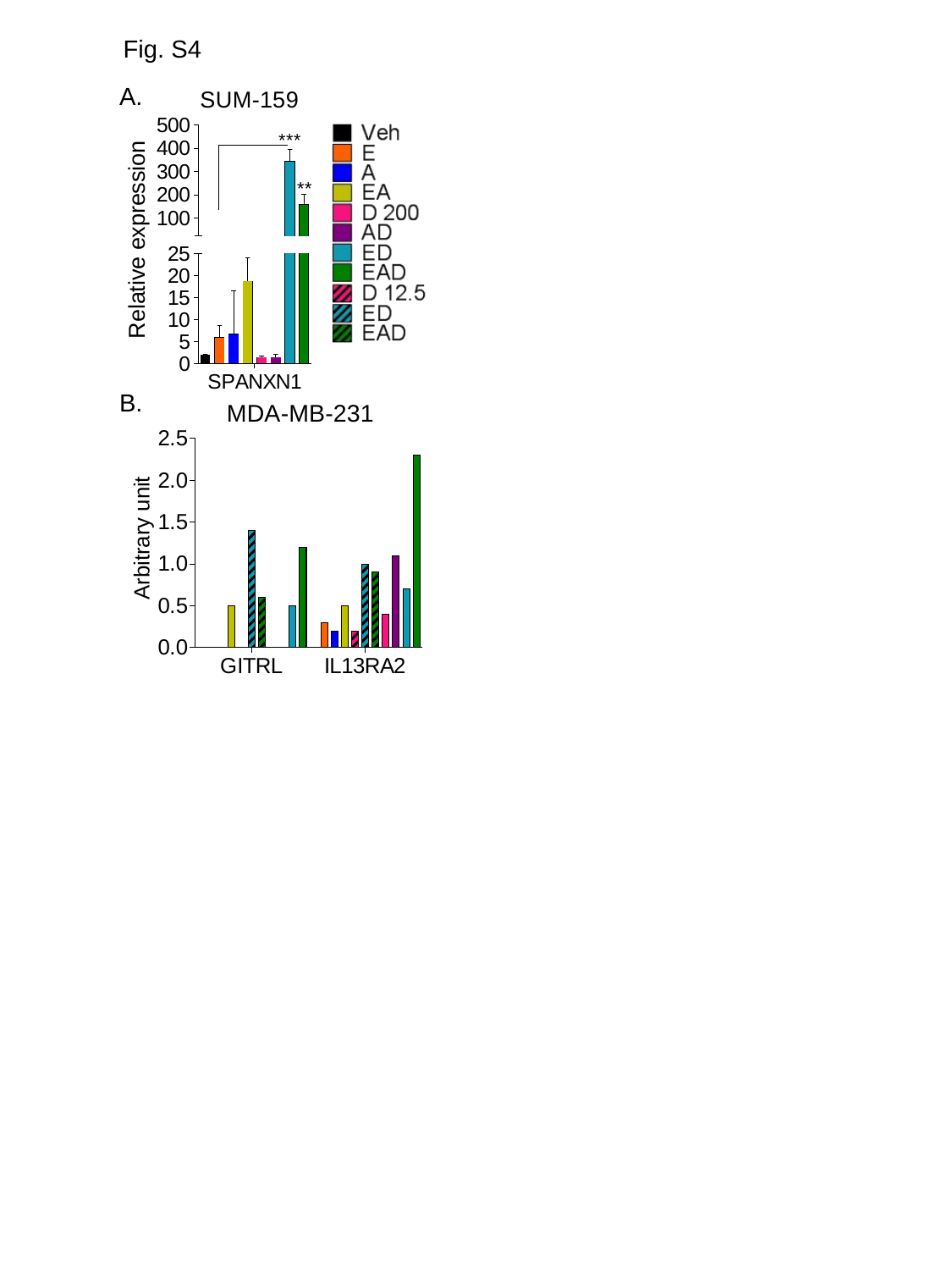

Fig. S4
A.
B.

Supplement: Supplementary file 12 — Figure S4. ED regulates expression of inflammatory genes. (A) qRT-PCR of SPANXN1 in SUM-159 cells treated as described in text (doxorubicin 200 nM). t test used to compare mean level of mRNA expression (± SEM) after RPL39 normalization. **p < 0.01, ***p < 0.001. (B) ImageJ quantification of GITRL, IL13RA2, and housekeeping β-actin protein in MDA-MB-231 cells treated as described (doxorubicin 12.5 and 200 nM). (PPTX 74 kb) [file 13058_2018_1068_MOESM12_ESM.pptx]

## Slide 1
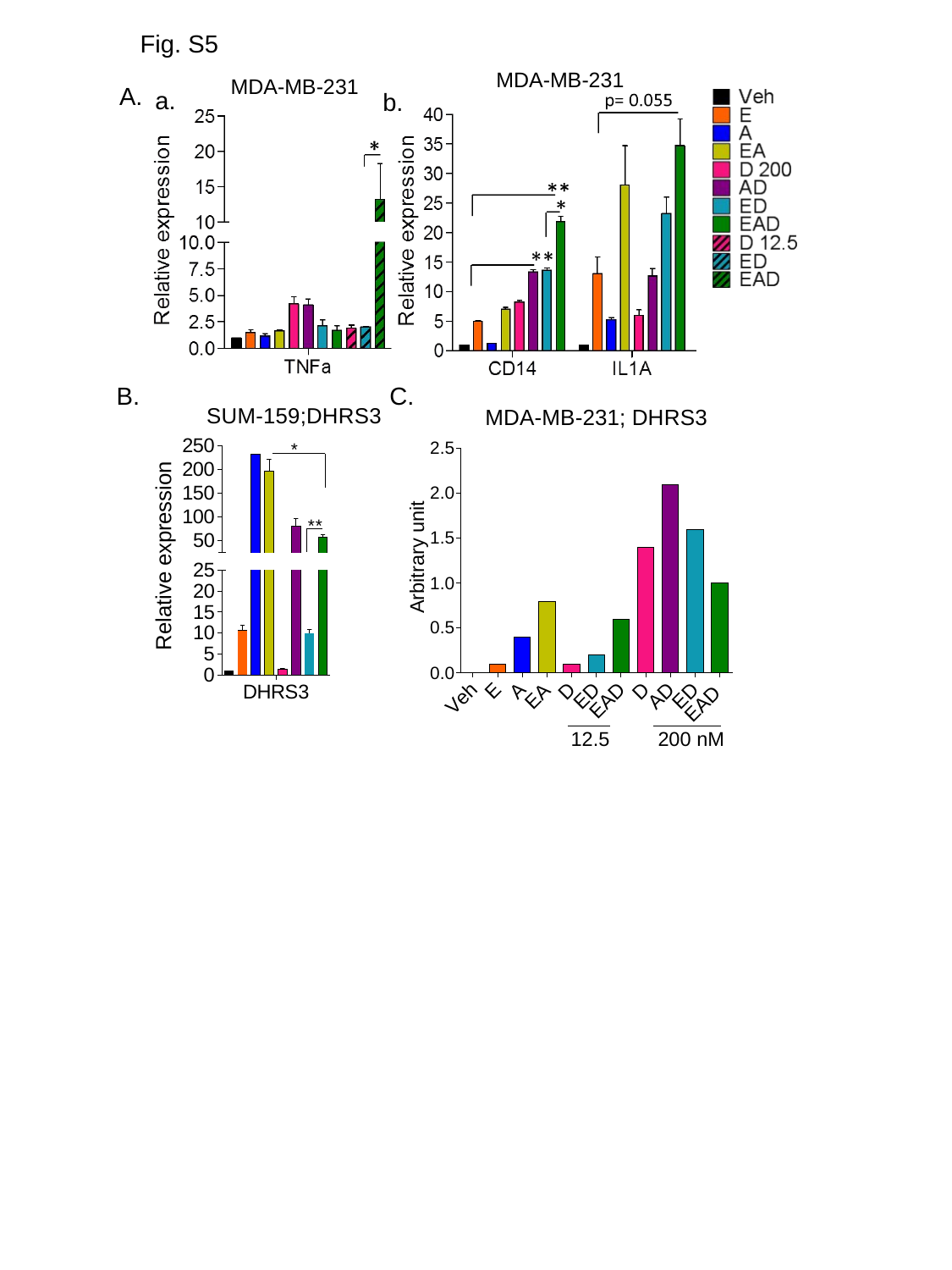

Fig. S5
MDA-MB-231
MDA-MB-231
A.
a.
b.
B.
C.

Supplement: Supplementary file 14 — Figure S5. genesEAD regulates inflammatory genes. qRT-PCR of TNF-α (a) and CD14 and IL1a (b) in MDA-MB-231 cells (A) and DHRS3 in SUM-159 cells (B) treated as described in text (doxorubicin 12.5 and 200 nM). t test used to compare mean level of mRNA expression (± SEM) after RPL39 normalization. *p < 0.05, **p < 0.01. (C) ImageJ quantification of DHRS3 and housekeeping β-actin proteins in MDA-MB-231 cells treated as described. (PPTX 105 kb) [file 13058_2018_1068_MOESM14_ESM.pptx]

## Slide 1
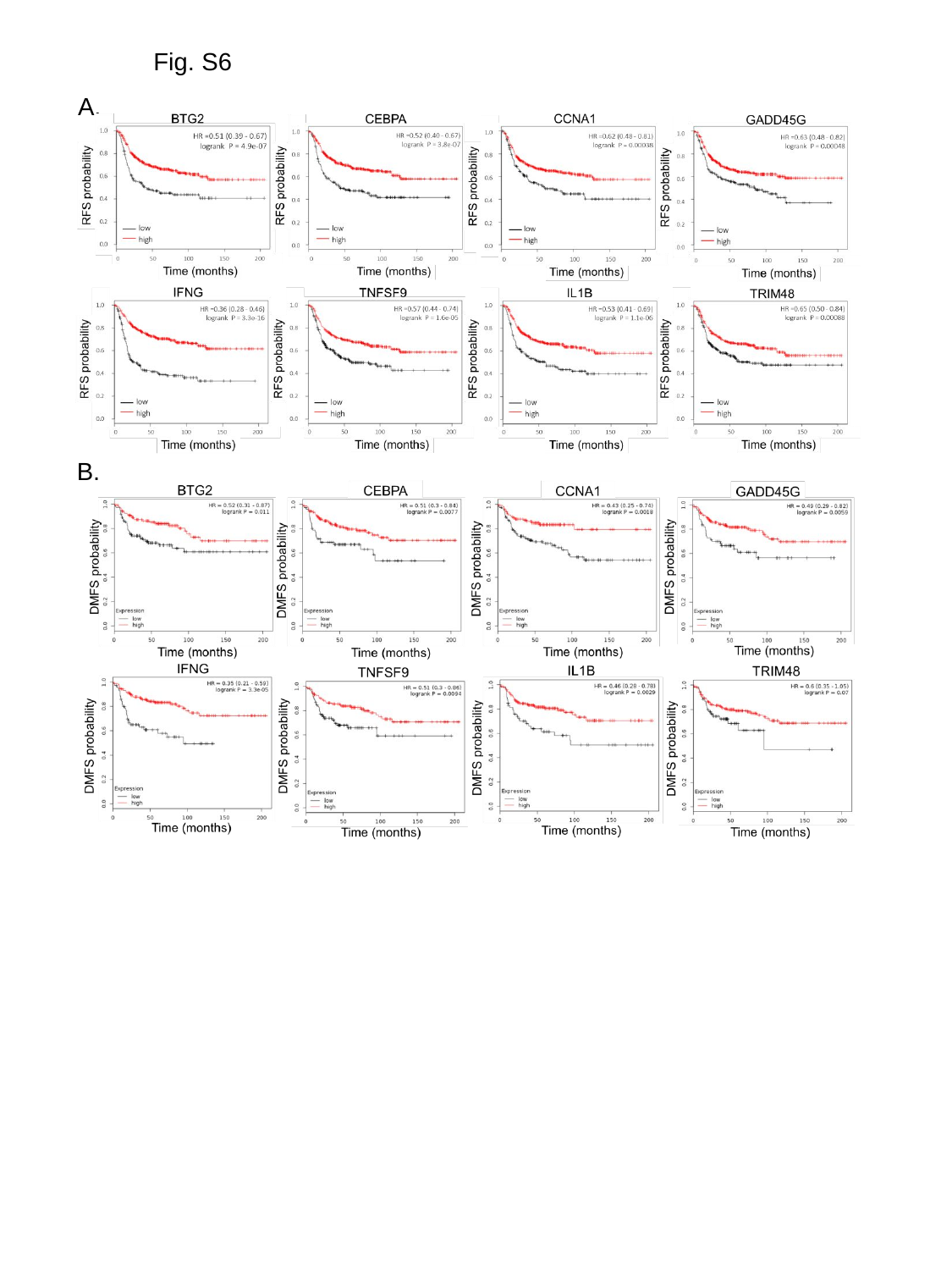

Fig. S6
A.
B.

Supplement: Supplementary file 15 — Figure S6. ED-induced genes correlate with a better prognosis in TNBC patients. Kaplan–Meier curves of relapsefree survival (RFS) (A) and metastases-free survival (DMFS) (B) showing correlation of ED-induced gene expression and prognosis in basal/TNBC patients, over a period of 12 years. (PPTX 378 kb) [file 13058_2018_1068_MOESM15_ESM.pptx]
