# Supplementary material for: Induction of cell cycle arrest and inflammatory genes by combined treatment with epigenetic, differentiating, and chemotherapeutic agents in triple-negative breast cancer
Source: Breast Cancer Res. 2018 Nov 28;20:145. doi: 10.1186/s13058-018-1068-x (PMC6263070; doi:10.1186/s13058-018-1068-x)
Supplement: Supplementary file 3 — Table S1. ED genes in comparison to entinostat and doxorubicin treatments. (DOCX 41 kb) [file 13058_2018_1068_MOESM3_ESM.docx]

|  | **ID** | **logFC** | **AveExpr** | **t** | **P.Value** | **adj.P.Val** | **B** |
| --- | --- | --- | --- | --- | --- | --- | --- |
| **4749** | CYP27B1 | 2.182 | 7.548 | 22.979 | 1.25E-11 | 4.33E-07 | 14.721 |
| **25082** | MMP7 | 1.769 | 7.051 | 20.200 | 6.04E-11 | 1.05E-06 | 13.777 |
| **25947** | NLF2 | 2.033 | 6.970 | 17.805 | 2.80E-10 | 3.24E-06 | 12.761 |
| **30162** | SILV | 1.789 | 7.337 | 16.867 | 5.38E-10 | 4.67E-06 | 12.299 |
| **2391** | C20ORF160 | 3.084 | 7.212 | 16.284 | 8.22E-10 | 5.70E-06 | 11.992 |
| **29898** | SEPX1 | 1.359 | 9.469 | 15.600 | 1.37E-09 | 7.07E-06 | 11.609 |
| **32867** | TSC22D3 | 1.356 | 7.220 | 15.551 | 1.43E-09 | 7.07E-06 | 11.581 |
| **2851** | C7ORF57 | 1.298 | 6.934 | 14.416 | 3.53E-09 | 1.29E-05 | 10.885 |
| **29002** | RGS16 | 2.091 | 6.956 | 14.382 | 3.62E-09 | 1.29E-05 | 10.863 |
| **2212** | C1ORF106 | 1.673 | 7.346 | 14.354 | 3.71E-09 | 1.29E-05 | 10.845 |
| **32181** | TM7SF2 | 1.744 | 8.029 | 13.624 | 6.88E-09 | 2.17E-05 | 10.352 |
| **6227** | FAM117A | 1.077 | 6.755 | 13.435 | 8.12E-09 | 2.35E-05 | 10.219 |
| **302** | ADC | 1.882 | 7.510 | 13.128 | 1.07E-08 | 2.84E-05 | 9.997 |
| **5898** | EPOR | 1.987 | 7.253 | 11.832 | 3.59E-08 | 8.89E-05 | 8.981 |
| **7498** | GADD45G | 2.389 | 7.327 | 11.758 | 3.86E-08 | 8.92E-05 | 8.919 |
| **8398** | HCP5 | 3.068 | 8.340 | 11.644 | 4.31E-08 | 9.35E-05 | 8.822 |
| **4661** | CXCR4 | 1.233 | 6.735 | 11.502 | 4.97E-08 | 9.79E-05 | 8.700 |
| **34214** | ZNF275 | 1.050 | 7.395 | 11.439 | 5.29E-08 | 9.79E-05 | 8.646 |
| **5094** | DHDH | 2.117 | 7.445 | 11.427 | 5.36E-08 | 9.79E-05 | 8.635 |
| **4381** | CRABP2 | 1.612 | 10.117 | 11.211 | 6.68E-08 | 0.000116 | 8.444 |
| **23676** | LRRC32 | 1.779 | 6.904 | 11.148 | 7.13E-08 | 0.000118 | 8.388 |
| **8209** | GSTM3 | 2.677 | 8.185 | 11.020 | 8.13E-08 | 0.000124 | 8.272 |
| **8335** | HAND1 | 3.187 | 7.779 | 10.929 | 8.94E-08 | 0.000129 | 8.189 |
| **3714** | CEBPA | 2.643 | 8.087 | 10.812 | 1.01E-07 | 0.00014 | 8.081 |
| **1684** | C10ORF10 | 1.634 | 7.009 | 10.638 | 1.22E-07 | 0.000162 | 7.918 |
| **16611** | LOC387763 | 1.226 | 7.115 | 10.569 | 1.31E-07 | 0.000168 | 7.853 |
| **23617** | LRAT | 1.120 | 6.969 | 10.447 | 1.49E-07 | 0.000185 | 7.736 |
| **4652** | CXCL16 | 1.455 | 7.285 | 10.200 | 1.96E-07 | 0.000227 | 7.494 |
| **6765** | FGFR3 | 1.679 | 7.483 | 10.197 | 1.97E-07 | 0.000227 | 7.490 |
| **29643** | SBK1 | 1.114 | 6.928 | 9.962 | 2.55E-07 | 0.000286 | 7.255 |
| **4817** | DACT3 | 2.228 | 7.597 | 9.867 | 2.85E-07 | 0.000308 | 7.158 |
| **4605** | CTSL2 | 3.564 | 8.454 | 9.699 | 3.45E-07 | 0.000361 | 6.984 |
| **12157** | HSPB8 | 2.732 | 8.246 | 9.676 | 3.54E-07 | 0.000361 | 6.959 |
| **819** | APLN | 1.209 | 6.857 | 9.452 | 4.59E-07 | 0.000409 | 6.723 |
| **19384** | LOC645558 | 3.113 | 7.454 | 9.452 | 4.59E-07 | 0.000409 | 6.723 |
| **3969** | CITED4 | 1.401 | 7.115 | 9.119 | 6.83E-07 | 0.000565 | 6.358 |
| **29006** | RGS2 | 1.684 | 9.213 | 8.904 | 8.88E-07 | 0.000689 | 6.116 |
| **33976** | ZCCHC12 | 4.246 | 7.575 | 8.890 | 9.03E-07 | 0.000689 | 6.101 |
| **29879** | SEPHS2 | 1.129 | 8.860 | 8.811 | 9.96E-07 | 0.000735 | 6.010 |
| **7407** | FUCA1 | 2.339 | 8.776 | 8.644 | 1.23E-06 | 0.000858 | 5.817 |
| **4925** | DDIT4L | 2.729 | 8.075 | 8.638 | 1.24E-06 | 0.000858 | 5.809 |
| **27447** | PI3 | 1.737 | 6.727 | 8.600 | 1.30E-06 | 0.000882 | 5.764 |
| **785** | AP3B2 | 1.048 | 6.732 | 8.508 | 1.46E-06 | 0.000945 | 5.656 |
| **872** | AQP3 | 1.010 | 6.797 | 8.417 | 1.64E-06 | 0.000979 | 5.547 |
| **5331** | DNASE2 | 1.097 | 8.778 | 8.370 | 1.74E-06 | 0.001005 | 5.491 |
| **22023** | LOC653506 | 1.710 | 8.123 | 8.304 | 1.89E-06 | 0.001065 | 5.412 |
| **29649** | SC5DL | 1.239 | 9.002 | 8.255 | 2.02E-06 | 0.001093 | 5.352 |
| **3967** | CITED1 | 1.358 | 7.240 | 8.195 | 2.18E-06 | 0.00114 | 5.278 |
| **12245** | IDH1 | 1.276 | 8.706 | 8.062 | 2.60E-06 | 0.00122 | 5.113 |
| **8216** | GSTT2 | 1.422 | 8.172 | 8.029 | 2.71E-06 | 0.00122 | 5.073 |
| **7754** | GJB2 | 1.344 | 8.585 | 8.015 | 2.76E-06 | 0.00122 | 5.055 |
| **27583** | PLA2G15 | 1.156 | 7.480 | 8.013 | 2.77E-06 | 0.00122 | 5.052 |
| **65** | ABCC5 | 1.436 | 7.673 | 8.011 | 2.78E-06 | 0.00122 | 5.050 |
| **2709** | C5ORF46 | 1.076 | 6.908 | 7.959 | 2.98E-06 | 0.001247 | 4.984 |
| **33599** | VWF | 1.127 | 6.644 | 7.946 | 3.03E-06 | 0.001247 | 4.968 |
| **28386** | PTCHD2 | 1.057 | 6.799 | 7.940 | 3.06E-06 | 0.001247 | 4.960 |
| **6534** | FAM90A7 | 2.272 | 6.911 | 7.909 | 3.18E-06 | 0.001284 | 4.921 |
| **29713** | SCIN | 2.033 | 7.776 | 7.883 | 3.30E-06 | 0.001315 | 4.888 |
| **31270** | SPRYD5 | 3.071 | 7.396 | 7.741 | 3.99E-06 | 0.001431 | 4.706 |
| **17030** | LOC399939 | 1.333 | 6.849 | 7.725 | 4.08E-06 | 0.001433 | 4.685 |
| **7383** | FSTL3 | 1.933 | 8.003 | 7.724 | 4.09E-06 | 0.001433 | 4.684 |
| **26234** | NUDT16L1 | 1.118 | 7.654 | 7.675 | 4.37E-06 | 0.001502 | 4.620 |
| **1446** | BCL6 | 1.245 | 7.911 | 7.624 | 4.68E-06 | 0.001544 | 4.555 |
| **4001** | CLDN1 | 1.844 | 7.921 | 7.608 | 4.79E-06 | 0.001544 | 4.533 |
| **4370** | CPT1C | 1.328 | 8.173 | 7.605 | 4.81E-06 | 0.001544 | 4.530 |
| **5868** | EPB41L5 | 1.219 | 7.331 | 7.583 | 4.96E-06 | 0.001546 | 4.501 |
| **4746** | CYP26B1 | 1.133 | 8.224 | 7.572 | 5.04E-06 | 0.001546 | 4.486 |
| **25496** | MYLIP | 1.080 | 9.355 | 7.535 | 5.30E-06 | 0.001605 | 4.438 |
| **2620** | C3ORF54 | 1.028 | 7.669 | 7.532 | 5.32E-06 | 0.001605 | 4.434 |
| **5235** | DLX3 | 1.296 | 7.027 | 7.520 | 5.41E-06 | 0.001618 | 4.417 |
| **30400** | SLC30A3 | 1.557 | 8.179 | 7.455 | 5.92E-06 | 0.001755 | 4.332 |
| **6451** | FAM64A | 1.291 | 9.064 | 7.413 | 6.28E-06 | 0.001807 | 4.276 |
| **7345** | FRG2B | 1.178 | 6.692 | 7.408 | 6.32E-06 | 0.001807 | 4.268 |
| **22962** | LOC729384 | 1.169 | 6.623 | 7.404 | 6.35E-06 | 0.001807 | 4.264 |
| **6937** | FLJ25404 | 1.227 | 7.332 | 7.296 | 7.40E-06 | 0.001991 | 4.118 |
| **29446** | RRAD | 2.359 | 7.603 | 7.276 | 7.61E-06 | 0.001994 | 4.092 |
| **6604** | FBXL15 | 1.090 | 7.882 | 7.273 | 7.64E-06 | 0.001994 | 4.087 |
| **1877** | C13ORF15 | 1.765 | 9.054 | 7.252 | 7.87E-06 | 0.002038 | 4.059 |
| **5591** | ECM1 | 1.101 | 7.541 | 7.194 | 8.55E-06 | 0.002164 | 3.980 |
| **6530** | FAM90A2P | 2.041 | 7.031 | 7.165 | 8.91E-06 | 0.002223 | 3.940 |
| **1656** | BTG2 | 1.360 | 7.025 | 7.126 | 9.43E-06 | 0.002303 | 3.886 |
| **29837** | SELENBP1 | 1.476 | 7.653 | 7.115 | 9.58E-06 | 0.002324 | 3.871 |
| **32910** | TSPAN33 | 1.193 | 7.104 | 7.097 | 9.83E-06 | 0.002369 | 3.846 |
| **4422** | CRISPLD2 | 1.855 | 8.552 | 7.035 | 1.07E-05 | 0.002518 | 3.760 |
| **27509** | PIM2 | 1.510 | 8.607 | 7.016 | 1.10E-05 | 0.002555 | 3.734 |
| **29136** | RND1 | 2.417 | 7.245 | 6.990 | 1.15E-05 | 0.002617 | 3.698 |
| **26146** | NRGN | 1.010 | 9.133 | 6.902 | 1.31E-05 | 0.002847 | 3.573 |
| **140** | ACBD7 | 1.102 | 7.793 | 6.856 | 1.39E-05 | 0.00295 | 3.509 |
| **31485** | STOM | 1.204 | 9.132 | 6.838 | 1.43E-05 | 0.003015 | 3.483 |
| **28710** | RAP1GAP | 1.612 | 7.567 | 6.786 | 1.55E-05 | 0.003104 | 3.409 |
| **26081** | NPL | 1.202 | 7.061 | 6.738 | 1.66E-05 | 0.003246 | 3.340 |
| **33051** | TUBA4A | 1.488 | 10.777 | 6.710 | 1.73E-05 | 0.003262 | 3.300 |
| **29948** | SERPINI1 | 1.502 | 7.690 | 6.657 | 1.87E-05 | 0.003477 | 3.224 |
| **32161** | TLR5 | 1.243 | 7.274 | 6.636 | 1.93E-05 | 0.003532 | 3.193 |
| **33466** | VASH2 | 1.574 | 7.301 | 6.619 | 1.98E-05 | 0.003604 | 3.169 |
| **30255** | SLC16A6 | 1.036 | 7.083 | 6.549 | 2.20E-05 | 0.003942 | 3.067 |
| **99** | ABHD8 | 1.325 | 9.697 | 6.466 | 2.50E-05 | 0.004235 | 2.944 |
| **6408** | FAM43A | 1.199 | 7.708 | 6.453 | 2.55E-05 | 0.004235 | 2.925 |
| **3123** | CAPN5 | 1.359 | 7.989 | 6.425 | 2.66E-05 | 0.004337 | 2.883 |
| **9260** | HS.193406 | 1.031 | 7.264 | 6.408 | 2.73E-05 | 0.004427 | 2.859 |
| **887** | ARC | 1.442 | 6.819 | 6.374 | 2.88E-05 | 0.00458 | 2.808 |
| **12746** | JUP | 1.139 | 7.363 | 6.340 | 3.03E-05 | 0.004771 | 2.757 |
| **33467** | VASN | 3.034 | 8.472 | 6.323 | 3.11E-05 | 0.004845 | 2.731 |
| **5783** | ELOVL3 | 1.469 | 7.292 | 6.315 | 3.15E-05 | 0.004879 | 2.720 |
| **25613** | NAT1 | 1.579 | 7.387 | 6.300 | 3.23E-05 | 0.004911 | 2.697 |
| **8775** | HPCAL4 | 1.645 | 7.085 | 6.230 | 3.60E-05 | 0.005225 | 2.591 |
| **33482** | VCX | 1.448 | 6.876 | 6.227 | 3.62E-05 | 0.005225 | 2.586 |
| **8762** | HOXD1 | 1.555 | 8.693 | 6.212 | 3.70E-05 | 0.00523 | 2.565 |
| **31643** | SYT11 | 2.335 | 8.806 | 6.194 | 3.80E-05 | 0.005254 | 2.537 |
| **16507** | LOC340970 | 2.246 | 6.974 | 6.177 | 3.91E-05 | 0.005274 | 2.511 |
| **34675** | ZSWIM3 | 1.010 | 7.157 | 6.169 | 3.96E-05 | 0.00532 | 2.499 |
| **26861** | OVOL2 | 1.228 | 6.827 | 6.124 | 4.25E-05 | 0.005601 | 2.430 |
| **32546** | TNFSF9 | 1.883 | 8.179 | 6.111 | 4.33E-05 | 0.005671 | 2.410 |
| **12570** | IQCD | 2.018 | 7.183 | 6.102 | 4.39E-05 | 0.005683 | 2.397 |
| **28670** | RAET1G | 1.070 | 7.285 | 6.089 | 4.48E-05 | 0.005738 | 2.377 |
| **26045** | NOV | 1.134 | 8.145 | 6.064 | 4.66E-05 | 0.005869 | 2.339 |
| **28853** | RBPMS2 | 1.277 | 7.738 | 6.058 | 4.71E-05 | 0.0059 | 2.328 |
| **4288** | CORO1A | 1.149 | 7.177 | 6.051 | 4.76E-05 | 0.005926 | 2.319 |
| **3288** | CCDC151 | 1.126 | 7.274 | 6.040 | 4.84E-05 | 0.005959 | 2.301 |
| **12099** | HSD11B2 | 1.030 | 6.748 | 6.015 | 5.04E-05 | 0.006007 | 2.263 |
| **27250** | PDLIM3 | 1.316 | 8.183 | 5.987 | 5.27E-05 | 0.006172 | 2.219 |
| **33056** | TUBB2A | 1.723 | 9.517 | 5.983 | 5.30E-05 | 0.006172 | 2.213 |
| **30693** | SNAPC2 | 1.176 | 9.131 | 5.977 | 5.35E-05 | 0.006211 | 2.204 |
| **3668** | CDKN1C | 1.202 | 7.181 | 5.964 | 5.46E-05 | 0.006244 | 2.184 |
| **25436** | MX1 | 1.371 | 6.962 | 5.962 | 5.48E-05 | 0.006244 | 2.180 |
| **22565** | LOC728393 | 1.505 | 6.808 | 5.961 | 5.49E-05 | 0.006244 | 2.178 |
| **9997** | HS.505676 | 1.064 | 8.862 | 5.957 | 5.53E-05 | 0.006244 | 2.172 |
| **7316** | FOXO4 | 2.071 | 8.333 | 5.953 | 5.56E-05 | 0.006244 | 2.167 |
| **6038** | ERN1 | 1.840 | 8.303 | 5.953 | 5.56E-05 | 0.006244 | 2.167 |
| **12264** | IFI27 | 1.389 | 7.457 | 5.948 | 5.61E-05 | 0.006244 | 2.158 |
| **25439** | MXD3 | 1.039 | 7.122 | 5.942 | 5.66E-05 | 0.006275 | 2.149 |
| **31359** | SSTR2 | 1.173 | 7.920 | 5.907 | 5.99E-05 | 0.00653 | 2.095 |
| **6518** | FAM90A1 | 1.597 | 6.759 | 5.890 | 6.15E-05 | 0.006669 | 2.068 |
| **3398** | CCL2 | 1.055 | 6.659 | 5.886 | 6.19E-05 | 0.006691 | 2.062 |
| **12141** | HSPA1A | 2.119 | 9.167 | 5.876 | 6.29E-05 | 0.00678 | 2.046 |
| **4647** | CXCL10 | 2.423 | 7.487 | 5.857 | 6.48E-05 | 0.00689 | 2.017 |
| **30246** | SLC16A10 | 2.418 | 8.059 | 5.811 | 6.98E-05 | 0.007169 | 1.944 |
| **6521** | FAM90A12 | 1.043 | 6.683 | 5.802 | 7.09E-05 | 0.007209 | 1.930 |
| **7276** | FOXA1 | 1.000 | 6.974 | 5.794 | 7.18E-05 | 0.007271 | 1.917 |
| **13060** | KIAA1602 | 1.494 | 7.879 | 5.731 | 7.94E-05 | 0.007741 | 1.818 |
| **30180** | SIRT4 | 1.472 | 7.228 | 5.719 | 8.10E-05 | 0.007849 | 1.799 |
| **1373** | BAMBI | 1.662 | 10.857 | 5.659 | 8.93E-05 | 0.008394 | 1.703 |
| **28756** | RASL11B | 1.552 | 7.147 | 5.645 | 9.15E-05 | 0.008555 | 1.680 |
| **3007** | CA2 | 1.692 | 8.065 | 5.618 | 9.56E-05 | 0.008864 | 1.637 |
| **4739** | CYP1A1 | 1.318 | 7.936 | 5.606 | 9.74E-05 | 0.008989 | 1.618 |
| **7337** | FRAT2 | 1.097 | 9.334 | 5.584 | 0.000101 | 0.0092 | 1.582 |
| **4981** | DEDD2 | 1.164 | 10.407 | 5.567 | 0.000104 | 0.009393 | 1.555 |
| **15710** | LOC100134006 | 1.145 | 6.672 | 5.508 | 0.000115 | 0.010059 | 1.459 |
| **3682** | CDR2L | 1.005 | 10.737 | 5.486 | 0.000119 | 0.010337 | 1.423 |
| **17164** | LOC401447 | 1.001 | 6.725 | 5.472 | 0.000122 | 0.010443 | 1.401 |
| **81** | ABCG4 | 1.232 | 7.255 | 5.456 | 0.000125 | 0.010693 | 1.375 |
| **31446** | STC1 | 1.383 | 9.900 | 5.437 | 0.000129 | 0.010935 | 1.344 |
| **29854** | SEMA3G | 1.067 | 6.518 | 5.433 | 0.00013 | 0.010945 | 1.338 |
| **6416** | FAM46C | 1.283 | 8.829 | 5.381 | 0.000142 | 0.011609 | 1.252 |
| **4057** | CLIC3 | 1.720 | 7.301 | 5.312 | 0.000159 | 0.012509 | 1.138 |
| **17279** | LOC440040 | 1.246 | 6.822 | 5.308 | 0.00016 | 0.01254 | 1.132 |
| **7790** | GLIPR2 | 1.146 | 8.172 | 5.303 | 0.000161 | 0.012594 | 1.124 |
| **33486** | VCX-C | 1.341 | 6.901 | 5.299 | 0.000162 | 0.012628 | 1.117 |
| **27247** | PDK4 | 1.138 | 7.526 | 5.271 | 0.00017 | 0.012953 | 1.070 |
| **6208** | FAM109A | 1.791 | 7.905 | 5.210 | 0.000189 | 0.013904 | 0.969 |
| **31148** | SPANXN1 | 1.483 | 6.777 | 5.206 | 0.00019 | 0.013948 | 0.963 |
| **28065** | PRAMEF9 | 2.431 | 7.136 | 5.200 | 0.000192 | 0.014019 | 0.953 |
| **28114** | PRIC285 | 1.062 | 8.458 | 5.178 | 0.000199 | 0.014395 | 0.916 |
| **4911** | DCXR | 1.057 | 9.554 | 5.141 | 0.000212 | 0.014756 | 0.854 |
| **12449** | IL32 | 1.618 | 7.330 | 5.133 | 0.000215 | 0.014911 | 0.840 |
| **3477** | CD14 | 1.457 | 8.517 | 5.118 | 0.000221 | 0.01513 | 0.816 |
| **2122** | C17ORF96 | 1.093 | 8.700 | 5.117 | 0.000221 | 0.015137 | 0.813 |
| **29011** | RGS4 | 1.728 | 8.834 | 5.110 | 0.000224 | 0.015214 | 0.802 |
| **28741** | RASD2 | 1.539 | 7.226 | 5.091 | 0.000231 | 0.01566 | 0.769 |
| **4128** | CNFN | 1.071 | 7.730 | 5.073 | 0.000239 | 0.016008 | 0.739 |
| **26928** | PADI4 | 1.444 | 6.928 | 5.055 | 0.000246 | 0.016218 | 0.709 |
| **4405** | CRELD2 | 1.132 | 8.933 | 5.050 | 0.000248 | 0.016242 | 0.701 |
| **14161** | LOC100129236 | 1.614 | 6.772 | 5.006 | 0.000267 | 0.017075 | 0.626 |
| **25942** | NKX3-1 | 1.149 | 9.344 | 5.006 | 0.000268 | 0.017075 | 0.625 |
| **14811** | LOC100131392 | 1.247 | 6.677 | 4.992 | 0.000274 | 0.017367 | 0.602 |
| **24171** | MED29 | 1.691 | 9.944 | 4.973 | 0.000283 | 0.017758 | 0.569 |
| **2052** | C16ORF80 | 1.129 | 9.760 | 4.961 | 0.000289 | 0.017952 | 0.549 |
| **3688** | CDS1 | 1.002 | 7.566 | 4.958 | 0.000291 | 0.018006 | 0.545 |
| **2998** | C9ORF95 | 1.130 | 7.414 | 4.954 | 0.000293 | 0.018092 | 0.537 |
| **32899** | TSPAN13 | 1.439 | 8.605 | 4.952 | 0.000294 | 0.018105 | 0.534 |
| **7497** | GADD45B | 1.077 | 9.470 | 4.947 | 0.000296 | 0.018154 | 0.526 |
| **23861** | MAFB | 2.042 | 7.400 | 4.933 | 0.000304 | 0.018485 | 0.501 |
| **20083** | LOC647334 | 1.002 | 6.537 | 4.916 | 0.000313 | 0.018836 | 0.473 |
| **25096** | MNX1 | 1.378 | 7.917 | 4.913 | 0.000314 | 0.018892 | 0.468 |
| **12536** | INSM2 | 2.771 | 7.426 | 4.873 | 0.000337 | 0.019682 | 0.399 |
| **28559** | RAB11FIP1 | 1.191 | 8.098 | 4.844 | 0.000355 | 0.020142 | 0.349 |
| **29150** | RNF122 | 1.137 | 7.216 | 4.826 | 0.000366 | 0.020401 | 0.318 |
| **30044** | SGK | 1.122 | 9.174 | 4.814 | 0.000374 | 0.020522 | 0.297 |
| **8661** | HMGCS1 | 1.755 | 8.830 | 4.775 | 0.0004 | 0.021578 | 0.231 |
| **6414** | FAM46A | 1.232 | 9.474 | 4.748 | 0.000419 | 0.022224 | 0.185 |
| **28957** | RFPL4A | 1.785 | 7.075 | 4.736 | 0.000428 | 0.022499 | 0.164 |
| **7625** | GCH1 | 1.548 | 8.200 | 4.732 | 0.000431 | 0.022577 | 0.156 |
| **6636** | FBXO32 | 1.219 | 8.359 | 4.714 | 0.000445 | 0.023105 | 0.125 |
| **20648** | LOC649346 | 1.951 | 6.933 | 4.691 | 0.000464 | 0.023562 | 0.085 |
| **3028** | CABYR | 2.103 | 7.532 | 4.689 | 0.000465 | 0.023562 | 0.082 |
| **8474** | HES6 | 1.552 | 8.555 | 4.661 | 0.000489 | 0.024251 | 0.032 |
| **28061** | PRAMEF5 | 1.451 | 6.820 | 4.653 | 0.000496 | 0.024521 | 0.018 |
| **34068** | ZFYVE21 | 1.005 | 9.180 | 4.647 | 0.000501 | 0.024707 | 0.008 |
| **27988** | PPP1R3C | 1.197 | 8.173 | 4.630 | 0.000516 | 0.02516 | -0.021 |
| **30045** | SGK1 | 1.204 | 8.889 | 4.624 | 0.000522 | 0.02535 | -0.032 |
| **16082** | LOC143666 | 1.160 | 8.199 | 4.617 | 0.000529 | 0.025518 | -0.044 |
| **24411** | MICB | 1.068 | 10.128 | 4.615 | 0.000531 | 0.02557 | -0.048 |
| **28060** | PRAMEF4 | 2.121 | 7.007 | 4.577 | 0.000568 | 0.026614 | -0.114 |
| **8217** | GSTT2B | 1.042 | 7.936 | 4.572 | 0.000573 | 0.026624 | -0.123 |
| **4522** | CSRP2 | 1.765 | 10.097 | 4.571 | 0.000574 | 0.026624 | -0.125 |
| **12271** | IFI6 | 1.115 | 9.088 | 4.563 | 0.000582 | 0.026698 | -0.138 |
| **32517** | TNFRSF10D | 1.649 | 8.081 | 4.535 | 0.000612 | 0.027342 | -0.187 |
| **7809** | GLS | 1.040 | 8.350 | 4.511 | 0.000638 | 0.028121 | -0.230 |
| **6536** | FAM90A9 | 1.051 | 6.741 | 4.507 | 0.000643 | 0.028204 | -0.237 |
| **22295** | LOC678655 | 1.282 | 7.756 | 4.505 | 0.000645 | 0.028221 | -0.240 |
| **5880** | EPHA4 | 1.464 | 7.837 | 4.483 | 0.000672 | 0.028888 | -0.280 |
| **32770** | TRIM48 | 1.433 | 6.961 | 4.478 | 0.000678 | 0.028993 | -0.288 |
| **26308** | OASL | 1.726 | 8.208 | 4.432 | 0.000736 | 0.030417 | -0.369 |
| **12600** | IRF7 | 2.128 | 8.418 | 4.428 | 0.000741 | 0.030464 | -0.376 |
| **25471** | MYH10 | 1.825 | 7.853 | 4.428 | 0.000741 | 0.030464 | -0.376 |
| **29079** | RIMS3 | 1.115 | 6.888 | 4.421 | 0.000751 | 0.030687 | -0.389 |
| **1488** | BEX2 | 1.383 | 7.633 | 4.418 | 0.000755 | 0.030806 | -0.394 |
| **1379** | BAPX1 | 1.046 | 7.954 | 4.417 | 0.000756 | 0.030811 | -0.396 |
| **15699** | LOC100133984 | 2.550 | 7.259 | 4.407 | 0.000769 | 0.030956 | -0.413 |
| **34308** | ZNF425 | 1.125 | 7.389 | 4.403 | 0.000775 | 0.031137 | -0.421 |
| **5673** | EGR2 | 1.485 | 7.490 | 4.386 | 0.000799 | 0.031783 | -0.451 |
| **22567** | LOC728400 | 1.629 | 6.923 | 4.372 | 0.00082 | 0.0321 | -0.476 |
| **3821** | CGN | 1.249 | 8.334 | 4.340 | 0.000869 | 0.033283 | -0.533 |
| **31152** | SPANXN5 | 2.250 | 7.007 | 4.335 | 0.000877 | 0.033503 | -0.542 |
| **5589** | ECHDC3 | 1.221 | 7.819 | 4.314 | 0.000911 | 0.034242 | -0.579 |
| **25995** | NMUR2 | 1.267 | 6.584 | 4.290 | 0.000951 | 0.035062 | -0.622 |
| **3840** | CHCHD7 | 1.338 | 8.176 | 4.258 | 0.001009 | 0.036337 | -0.680 |
| **656** | ANKRA2 | 1.135 | 7.637 | 4.245 | 0.001032 | 0.036785 | -0.702 |
| **1139** | ATF3 | 1.320 | 7.728 | 4.243 | 0.001037 | 0.036884 | -0.706 |
| **29767** | SDC4 | 1.086 | 9.151 | 4.226 | 0.001069 | 0.037716 | -0.737 |
| **28740** | RASD1 | 1.820 | 10.032 | 4.187 | 0.001148 | 0.039074 | -0.806 |
| **27146** | PCK2 | 1.049 | 7.545 | 4.182 | 0.001157 | 0.039274 | -0.814 |
| **15167** | LOC100132510 | 1.049 | 7.587 | 4.159 | 0.001207 | 0.039982 | -0.856 |
| **27418** | PHGDH | 1.171 | 7.326 | 4.156 | 0.001214 | 0.040039 | -0.862 |
| **3647** | CDK2AP2 | 1.279 | 8.334 | 4.151 | 0.001225 | 0.040107 | -0.871 |
| **30407** | SLC31A1 | 1.171 | 8.685 | 4.143 | 0.001245 | 0.040393 | -0.886 |
| **12407** | IL1A | 1.205 | 7.481 | 4.142 | 0.001246 | 0.040395 | -0.887 |
| **20937** | LOC650373 | 1.027 | 6.546 | 4.136 | 0.00126 | 0.040688 | -0.898 |
| **32655** | TPMT | 1.342 | 8.846 | 4.118 | 0.001303 | 0.041493 | -0.931 |
| **25825** | NEU1 | 2.013 | 9.338 | 4.117 | 0.001304 | 0.041509 | -0.932 |
| **8472** | HES4 | 1.227 | 10.548 | 4.085 | 0.001383 | 0.042962 | -0.990 |
| **20825** | LOC649987 | 1.026 | 7.149 | 4.082 | 0.001391 | 0.043098 | -0.995 |
| **34053** | ZFP90 | 1.101 | 8.185 | 4.065 | 0.001434 | 0.043979 | -1.025 |
| **3418** | CCNA1 | 1.840 | 7.396 | 4.026 | 0.001541 | 0.046174 | -1.096 |
| **25736** | NDRG1 | 1.151 | 9.995 | 4.007 | 0.001598 | 0.04726 | -1.131 |
| **22562** | LOC728379 | 1.011 | 6.627 | 3.964 | 0.001729 | 0.049528 | -1.209 |
| **12250** | IDI1 | 1.015 | 9.000 | 3.961 | 0.001738 | 0.049695 | -1.214 |
| **7684** | GFPT2 | 1.379 | 8.730 | 3.955 | 0.001758 | 0.049862 | -1.225 |
| **3425** | CCND1 | -1.010 | 11.895 | -4.009 | 0.00159 | 0.047141 | -1.127 |
| **26932** | PAFAH1B1 | -1.217 | 8.706 | -4.034 | 0.00152 | 0.045724 | -1.082 |
| **31822** | TBK1 | -1.022 | 8.255 | -4.091 | 0.001369 | 0.042638 | -0.980 |
| **32021** | TGFBR2 | -1.596 | 9.245 | -4.174 | 0.001175 | 0.039436 | -0.830 |
| **32592** | TOMM20 | -1.169 | 10.815 | -4.174 | 0.001174 | 0.039429 | -0.829 |
| **32527** | TNFRSF1A | -1.079 | 9.023 | -4.198 | 0.001124 | 0.038722 | -0.786 |
| **31265** | SPRY2 | -1.033 | 9.320 | -4.267 | 0.000991 | 0.035938 | -0.663 |
| **30319** | SLC25A13 | -1.130 | 8.609 | -4.334 | 0.000878 | 0.033503 | -0.543 |
| **5033** | DEK | -1.098 | 10.446 | -4.361 | 0.000836 | 0.032472 | -0.495 |
| **23911** | MAK16 | -1.010 | 8.840 | -4.375 | 0.000816 | 0.032046 | -0.471 |
| **32331** | TMEM185B | -1.001 | 7.925 | -4.381 | 0.000807 | 0.031859 | -0.460 |
| **22236** | LOC654103 | -1.133 | 9.187 | -4.449 | 0.000714 | 0.029992 | -0.340 |
| **5731** | EIF4B | -1.062 | 10.721 | -4.530 | 0.000618 | 0.027586 | -0.197 |
| **10850** | HS.551128 | -1.112 | 7.768 | -4.589 | 0.000556 | 0.026234 | -0.093 |
| **31241** | SPOP | -1.062 | 9.053 | -4.627 | 0.00052 | 0.025256 | -0.027 |
| **4588** | CTPS | -1.066 | 9.453 | -4.694 | 0.000461 | 0.023541 | 0.090 |
| **2038** | C16ORF63 | -1.002 | 8.542 | -4.718 | 0.000442 | 0.023032 | 0.132 |
| **30012** | SFRS4 | -1.142 | 9.837 | -4.741 | 0.000424 | 0.022374 | 0.172 |
| **30611** | SMAD3 | -1.081 | 8.963 | -4.771 | 0.000403 | 0.021685 | 0.223 |
| **3656** | CDK6 | -1.817 | 9.056 | -4.782 | 0.000395 | 0.021346 | 0.243 |
| **75** | ABCE1 | -1.023 | 9.249 | -4.994 | 0.000273 | 0.017329 | 0.605 |
| **28353** | PSMD7 | -1.005 | 9.500 | -5.125 | 0.000218 | 0.015044 | 0.827 |
| **27028** | PATL1 | -1.044 | 9.001 | -5.392 | 0.000139 | 0.011447 | 1.270 |
| **2918** | C9ORF114 | -1.067 | 8.897 | -5.424 | 0.000132 | 0.010988 | 1.322 |
| **29463** | RRP15 | -1.042 | 9.284 | -5.435 | 0.000129 | 0.010945 | 1.340 |
| **28436** | PTPN11 | -1.250 | 9.822 | -5.501 | 0.000116 | 0.01014 | 1.449 |
| **16221** | LOC203547 | -1.106 | 10.770 | -5.821 | 6.87E-05 | 0.007138 | 1.960 |
| **744** | ANTXR2 | -1.055 | 10.181 | -6.395 | 2.79E-05 | 0.004457 | 2.839 |
| **8234** | GTF2H2B | -1.160 | 8.126 | -6.792 | 1.53E-05 | 0.003104 | 3.418 |
| **33359** | URB2 | -1.088 | 8.079 | -7.859 | 3.41E-06 | 0.001328 | 4.857 |
