# Supplementary material for: Induction of cell cycle arrest and inflammatory genes by combined treatment with epigenetic, differentiating, and chemotherapeutic agents in triple-negative breast cancer
Source: Breast Cancer Res. 2018 Nov 28;20:145. doi: 10.1186/s13058-018-1068-x (PMC6263070; doi:10.1186/s13058-018-1068-x)
Supplement: Supplementary file 4 — Table S2. Genes differentially expressed by ED treatment and validated by qRT-PCR. (DOCX 14 kb) [file 13058_2018_1068_MOESM4_ESM.docx]

**Table S2. Genes differentially expressed by ED treatment and validated by qRT-PCR.**

| **ID** | **logFC** | **AveExpr** | **t** | **p-value** | **FDR** |
| --- | --- | --- | --- | --- | --- |
| **MMP7** | 1.8 | 7.1 | 20.2 | 6E-11 | 1E-06 |
| **SEPX1** | 1.4 | 9.5 | 15.6 | 1E-09 | 7E-06 |
| **GADD45G** | 2.4 | 7.3 | 11.8 | 4E-08 | 9E-05 |
| **CXCR4** | 1.2 | 6.7 | 11.5 | 5E-08 | 1E-04 |
| **CRABP2** | 1.6 | 10.1 | 11.2 | 7E-08 | 1E-04 |
| **GSTM3** | 2.7 | 8.2 | 11.0 | 8E-08 | 1E-04 |
| **CEBPA** | 2.6 | 8.1 | 10.8 | 1E-07 | 1E-04 |
| **LRAT** | 1.1 | 7.0 | 10.4 | 1E-07 | 2E-04 |
| **CXCL16** | 1.5 | 7.3 | 10.2 | 2E-07 | 2E-04 |
| **FGFR3** | 1.7 | 7.5 | 10.2 | 2E-07 | 2E-04 |
| **HSPB8** | 2.7 | 8.2 | 9.7 | 4E-07 | 4E-04 |
| **DDIT4L** | 2.7 | 8.1 | 8.6 | 1E-06 | 9E-04 |
| **AQP3** | 1.0 | 6.8 | 8.4 | 2E-06 | 1E-03 |
| **CITED1** | 1.4 | 7.2 | 8.2 | 2E-06 | 1E-03 |
| **SPRYD5** | 3.1 | 7.4 | 7.7 | 4E-06 | 1E-03 |
| **BCL6** | 1.2 | 7.9 | 7.6 | 5E-06 | 2E-03 |
| **CYP26B1** | 1.1 | 8.2 | 7.6 | 5E-06 | 2E-03 |
| **DLX3** | 1.3 | 7.0 | 7.5 | 5E-06 | 2E-03 |
| **BTG2** | 1.4 | 7.0 | 7.1 | 9E-06 | 2E-03 |
| **TLR5** | 1.2 | 7.3 | 6.6 | 2E-05 | 4E-03 |
| **HOXD1** | 1.6 | 8.7 | 6.2 | 4E-05 | 5E-03 |
| **OVOL2** | 1.2 | 6.8 | 6.1 | 4E-05 | 6E-03 |
| **TNFSF9** | 1.9 | 8.2 | 6.1 | 4E-05 | 6E-03 |
| **RAET1G** | 1.1 | 7.3 | 6.1 | 4E-05 | 6E-03 |
| **CDKN1C** | 1.2 | 7.2 | 6.0 | 5E-05 | 6E-03 |
| **FOXO4** | 2.1 | 8.3 | 6.0 | 6E-05 | 6E-03 |
| **CCL2** | 1.1 | 6.7 | 5.9 | 6E-05 | 7E-03 |
| **FOXA1** | 1.0 | 7.0 | 5.8 | 7E-05 | 7E-03 |
| **BAMBI** | 1.7 | 10.9 | 5.7 | 9E-05 | 8E-03 |
| **SPANXN1** | 1.5 | 6.8 | 5.2 | 2E-04 | 1E-02 |
| **IL32** | 1.6 | 7.3 | 5.1 | 2E-04 | 1E-02 |
| **CD14** | 1.5 | 8.5 | 5.1 | 2E-04 | 2E-02 |
| **PADI4** | 1.4 | 6.9 | 5.1 | 2E-04 | 2E-02 |
| **NKX3-1** | 1.1 | 9.3 | 5.0 | 3E-04 | 2E-02 |
| **TRIM48** | 1.4 | 7.0 | 4.5 | 7E-04 | 3E-02 |
| **EGR2** | 1.5 | 7.5 | 4.4 | 8E-04 | 3E-02 |
| **SPANXN5** | 2.3 | 7.0 | 4.3 | 9E-04 | 3E-02 |
| **IL1A** | 1.2 | 7.5 | 4.1 | 1E-03 | 4E-02 |
| **CCNA1** | 1.8 | 7.4 | 4.0 | 2E-03 | 5E-02 |
| **CCND1** | -1.0 | 11.9 | -4.0 | 2E-03 | 5E-02 |
| **CDK6** | -1.8 | 9.1 | -4.8 | 4E-04 | 2E-02 |

The expression of 65 genes induced by ED (out of 241, adjusted p value <0.01) was tested by qRT-PCR. 65% of these genes had a fold change in qRT-PCR higher than 1.3 and 37% higher than 2. Genes are sorted by t-statistic in the array. FC, array fold change; t, t-statistic from Limma; FDR, false discovery rate.
