# Supplementary material for: Induction of cell cycle arrest and inflammatory genes by combined treatment with epigenetic, differentiating, and chemotherapeutic agents in triple-negative breast cancer
Source: Breast Cancer Res. 2018 Nov 28;20:145. doi: 10.1186/s13058-018-1068-x (PMC6263070; doi:10.1186/s13058-018-1068-x)
Supplement: Supplementary file 5 — Table S3. Ingenuity® Pathway Analysis of ED genes (DOCX 13 kb) [file 13058_2018_1068_MOESM5_ESM.docx]

**Table S3. Ingenuity pathway analysis of ED genes.**

| **Top Networks** |  |  |
| --- | --- | --- |
| **Associated Network Functions** | Score |  |
| Infectious Disease, Antimicrobial Response, Inflammatory Response | 39 |  |
| Cancer, Neurological Disease, Infectious Disease | 33 |  |
| Cell-To-Cell Signaling and Interaction, Cellular Movement, Connective Tissue Development and Function | 33 |  |
| Hair and Skin Development and Function, Connective Tissue Development and Function, Tissue Morphology | 30 |  |
| Hereditary Disorder, Neurological Disease, Molecular Transport | 29 |  |
|  |  |  |
| **Top Canonical Pathways** |  |  |
| Name | p-value | Ratio |
| Agranulocyte Adhesion and Diapedesis | 7.56E-05 | 10 out of 18 |
| Granulocyte Adhesion and Diapedesis | 2.38E-04 | 9 out of 175 |
| Hepatic Fibrosis / Hepatic Stellate Cell Activation | 3.02E-04 | 8 out of 146 |
| Superpathway of Cholesterol Biosynthesis | 3.98E-04 | 4 out of 86 |
| GADD45 Signaling | 1.49E-03 | 3 out of 22 |
|  |  |  |
| **Top Bio Functions** |  |  |
| **Molecular and Cellular Functions** |  |  |
| Name | p-value | # Molecules |
| Cellular Development | 1.49E-10 -6.55E-03 | 93 |
| Cell Death and Survival | 5.57E-10 -6.63E-03 | 88 |
| Cellular Growth and Proliferation | 2.80E-07 -5.31E-03 | 86 |
| Cellular Movement | 9.15E-07 -6.79E-03 | 58 |
| Lipid Metabolism | 1.16E-06-5.01E-03 | 40 |
|  |  |  |
| **Physiological System Development and Function** |  |  |
| Name | p-value | # Molecules |
| Digestive System Development and Function | 5.90E-08 -6.46E-03 | 36 |
| Tissue Morphology | 3.09E-06 - 5.40E-03 | 57 |
| Hematological System Development and Function | 6.28E-06 -6.79E-03 | 68 |
| Hematopoiesis | 6.28E-06 -6.46E-03 | 33 |
| Tissue Development | 2.21E-05 - 6.79E-03 | 64 |

Functions and statistics associated to the most significant networks, canonical pathways, and biological functions following ED treatment are shown.
