# Supplementary material for: Induction of cell cycle arrest and inflammatory genes by combined treatment with epigenetic, differentiating, and chemotherapeutic agents in triple-negative breast cancer
Source: Breast Cancer Res. 2018 Nov 28;20:145. doi: 10.1186/s13058-018-1068-x (PMC6263070; doi:10.1186/s13058-018-1068-x)
Supplement: Supplementary file 6 — Table S4. Gene set analysis on ED genes. (DOCX 12 kb) [file 13058_2018_1068_MOESM6_ESM.docx]

**Table S4. Gene set analysis on ED genes.**

| Pathway | pval | FDR | genes |
| --- | --- | --- | --- |
| HALLMARK_TNFA_SIGNALING_VIA_NFKB | 0 | 0 | EGR3, ZC3H12A, SOD2, IL1A, BTG2, FOSL1, GCH1, CCL2, PHLDA1, TNFSF9, CXCL10, TNFRSF9, SGK1, ID2, CYR61, TUBB2A, FOS, CSF2, CCND1, CLCF1, FOSB, SLC16A6, BCL6, SMAD3, IFIH1, GFPT2 |
| HALLMARK_G2M_CHECKPOINT | 0 | 3.00E-05 | LMNB1, CCND1, KIF20B, CBX1, HIF1A, RAD23B, SMAD3, PAFAH1B1 |
| HALLMARK_INTERFERON_GAMMA_RESPONSE | 0.00031 | 0.00524 | ISG15, MX1, IRF7, CXCL10, OASL, IFIH1, IFI27, PRIC285, SOD2, GCH1, CCL2, HIF1A, HLA-B, ST3GAL5 |
| HALLMARK_INTERFERON_ALPHA_RESPONSE | 0.00051 | 0.0064 | MX1, ISG15, IRF7, IFITM1, IFI27, OASL, CXCL10, IFIH1, PRIC285 |
| HALLMARK_E2F_TARGETS | 0.00079 | 0.00774 | TIPIN, LMNB1, ASF1B, DEK, NBN, TP53, NUP153, MXD3 |
| HALLMARK_P53_PATHWAY | 0.00104 | 0.00774 | BTG2, TP53, RRAD, DCXR, FOS, AK1, TNFSF9, STOM, FUCA1, EPHX1, H2AFJ, RGS16, CEBPA, ANKRA2, VWA5A, VDR, ABCC5, IL1A, PMM1, RXRA, CSRNP2, TSPYL2, MXD4 |
| HALLMARK_MTORC1_SIGNALING | 0.00108 | 0.00774 | TUBA4A, CORO1A, IDH1, TM7SF2, BTG2, STC1, CTH, SC5DL, TBK1, GLRX, PHGDH, CXCR4 |
| HALLMARK_UV_RESPONSE_UP | 0.00238 | 0.01486 | NXF1, SLC6A8, TUBA4A, EPHX1, CTSL2, FOS, SLC25A4, CDKN1C, HLA-F, NAT1, RRAD, ACAA1, SOD2, PDLIM3, CYP1A1, FOSB, DNAJB1, GCH1, BTG2, PARP2, AQP3, CA2 |
| HALLMARK_APOPTOSIS | 0.00284 | 0.01577 | BIK, IL1A, EGR3, CCND1, BTG2, PTK2, GCH1, PLAT, SOD2, CD14, CTH, SC5DL, SLC20A1, CCNA1 |
| HALLMARK_UNFOLDED_PROTEIN_RESPONSE | 0.00903 | 0.04514 | WFS1, TSPYL2, ERN1, CCL2, EIF4A2, TUBB2A |
| HALLMARK_MITOTIC_SPINDLE | 0.01036 | 0.04709 | PAFAH1B1, KATNB1, NCK1, KIF20B, MYH10, TUBA4A, LMNB1 |

Results of gene set analysis comparing ED genes against single treatment entinostat and doxorubicin genes against MSigDB hallmark genesetes support observations from IPA analysis.
