# Supplementary material for: Induction of cell cycle arrest and inflammatory genes by combined treatment with epigenetic, differentiating, and chemotherapeutic agents in triple-negative breast cancer
Source: Breast Cancer Res. 2018 Nov 28;20:145. doi: 10.1186/s13058-018-1068-x (PMC6263070; doi:10.1186/s13058-018-1068-x)
Supplement: Supplementary file 8 — Table S5. ED and EAD induce growth arrest in HCC1937 TNBC cells. (DOCX 15 kb) [file 13058_2018_1068_MOESM8_ESM.docx]

**Table S5. ED and EAD induce growth arrest.**

Flow cytometry determination of the percentage of cell cycle distribution of HCC1937 treated with different groups containing doxorubicin 50 nM for 48h.
