# Supplementary material for: Induction of cell cycle arrest and inflammatory genes by combined treatment with epigenetic, differentiating, and chemotherapeutic agents in triple-negative breast cancer
Source: Breast Cancer Res. 2018 Nov 28;20:145. doi: 10.1186/s13058-018-1068-x (PMC6263070; doi:10.1186/s13058-018-1068-x)
Supplement: Supplementary file 9 — Table S6. IFN-α genes induced by ED in MDA-MB-231 cells and correlated with immune infiltration. (DOCX 14 kb) [file 13058_2018_1068_MOESM9_ESM.docx]

**Table S6. IFN-alpha genes regulated by ED.**

TRIM26

IFI30

TRAFD1

PARP14

TRIM21

IFIH1

CD74

PSMB8

HERC6

NCOA7

PARP12

IRF9

NMI

PRIC285

IFI35

TDRD7

OASL

IRF7

IFITM1

GMPR

EPSTI1

SP110

MOV10

RNF31

CD47

TRIM5

IFI44L

OGFR

GBP2

PARP9

CASP1

PLSCR1

LAMP3

PSMB9

IL15

IFI27

CXCL10

MX1

HLA-C

CASP8

C1S

DHX58

RSAD2

IRF2

OAS1

IL7

CSF1

BATF2

GBP4

TMEM140

USP18

IL4R

SAMD9L

CXCL11

TRIM14

RTP4

CNP

SELL

IRF1

UBE2L6

WARS

LGALS3BP

ADAR

RIPK2

IFI44

IFIT3

EIF2AK2

ELF1

TRIM25

NUB1

PROCR

ISG20

SAMD9

SLC25A28

LAP3

BST2

LY6E

PNPT1

FAM125A

IFIT2

TAP1

STAT2

FAM46A

IFITM2

IFITM3

TXNIP

ISG15

PSMA3

PSME1

B2M

PSME2
