# Supplementary material for: Induction of cell cycle arrest and inflammatory genes by combined treatment with epigenetic, differentiating, and chemotherapeutic agents in triple-negative breast cancer
Source: Breast Cancer Res. 2018 Nov 28;20:145. doi: 10.1186/s13058-018-1068-x (PMC6263070; doi:10.1186/s13058-018-1068-x)
Supplement: Supplementary file 11 — Table S7. IFN-γ genes induced by ED in MDA-MB-231 cells and correlated with immune infiltration. (DOCX 15 kb) [file 13058_2018_1068_MOESM11_ESM.docx]

**Table S7. IFN-gamma genes regulated by ED.**

CD40

TNFAIP2

CCL5

EPSTI1

IL6

SLAMF7

CASP1

CFB

STAT4

MX1

PTGS2

PELI1

ST3GAL5

IFI27

ICAM1

CXCL10

PIM1

IFI44L

DDX58

CFH

MVP

RNF31

OGFR

SRI

NCOA3

SP110

PSMB9

IL15

TNFAIP3

CASP7

FPR1

PLA2G4A

BPGM

OAS3

SOCS3

XAF1

OAS2

PTPN2

PLSCR1

ARL4A

CD74

IRF9

MYD88

TRIM26

TAPBP

IFI30

HLA-G

TRIM21

IFIH1

RBCK1

TRAFD1

PARP14

PSMB8

HERC6

RIPK1

PARP12

ZNFX1

NMI

CASP4

RAPGEF6

PSMA2

HIF1A

HLA-DMA

IFI35

PRIC285

OASL

ARID5B

TDRD7

HLA-B

SOD2

GCH1

IRF7

RSAD2

BANK1

FAS

IRF2

ZBP1

FGL2

LCP2

IRF4

IL15RA

IL18BP

ST8SIA4

IRF5

CASP8

TNFAIP6

KLRK1

CIITA

SECTM1

IL2RB

CD38

TNFSF10

MX2

DHX58

IL4R

ITGB7

LATS2

IFNAR2

USP18

VAMP5

SOCS1

METTL7B

IRF8

GBP4

AUTS2

RNF213

EIF4E3

SAMD9L

SSPN

VCAM1

CCL2

PDE4B

XCL1

CD69

SERPING1

CD274

GBP6

NOD1

IL7

CXCL11

TRIM14

P2RY14

PTPN6

RTP4

SAMHD1

APOL6

CD86

PML

NLRC5

C1S

JAK2

CSF2RB

BATF2

CMKLR1

HLA-DQA1

IL10RA

SELP

CXCL9

CCL7

GPR18

FCGR1A

BST2

LY6E

STAT1

NUP93

ISOC1

EIF2AK2

IFI44

UBE2L6

LGALS3BP

RIPK2

IRF1

WARS

UPP1

ISG20

LYSMD2

ADAR

SPPL2A

MTHFD2

STAT3

CASP3

IFIT1

TRIM25

IFIT3

SLC25A28

LAP3

PTPN1

IFIT2

NFKBIA

STAT2

TOR1B

TAP1

BTG1

PNPT1

PSMB10

NFKB1

PSMA3

VAMP8

PSME1

IFITM2

IFITM3

PSMB2

PFKP

TXNIP

CDKN1A

B2M

PSME2

HLA-A

ISG15

MT2A
