# Supplementary material for: Induction of cell cycle arrest and inflammatory genes by combined treatment with epigenetic, differentiating, and chemotherapeutic agents in triple-negative breast cancer
Source: Breast Cancer Res. 2018 Nov 28;20:145. doi: 10.1186/s13058-018-1068-x (PMC6263070; doi:10.1186/s13058-018-1068-x)
Supplement: Supplementary file 13 — Table S8. Inflammation score significance in mouse xenografts. (DOCX 14 kb) [file 13058_2018_1068_MOESM13_ESM.docx]

**Table S8. Inflammation scores significance in mouse xenografts.**

|  | **Inflammation** | |
| --- | --- | --- |
|  | Estimate | P. value |
| **Veh** | 0.017(0.001, 0.240) | 0.003 |
| **E** | NA | NA |
| **A** | 0.054(0.005, 0.539) | 0.013 |
| **AD** | NA | NA |
| **EA** | 0.017(0.001, 0.240) | 0.003 |
| **D** | 0.156(0.021, 1.175) | 0.071 |
| **ED** | 0.080(0.010, 0.646) | 0.018 |

Estimation and p value show the difference in inflammation content in tumor xenografts (n= 7-10/ group) from the indicated treatment versus the triple combination of entinostat, ATRA and doxorubicin (EAD). NA= not-available. Veh, vehicle.
